# Integrative characterization of human hepatocellular carcinoma‐derived organoids and their HBV‐associated biology

**DOI:** 10.1002/ctm2.70853

**Published:** 2026-10-06

**Authors:** Seo‐Hyeon Kim, Young Rok Choi, Eun Woo Choi, Se Hui Kim, Seung Cheol Oh, Jae Yoon Kim, Su Young Hong, Suk Kyun Hong, Kwang‐Woong Lee, Ja‐Lok Ku

**Affiliations:** ^1^ Cancer Research Institute Seoul National University College of Medicine Seoul South Korea; ^2^ Department of Biomedical Sciences Seoul National University College of Medicine Seoul South Korea; ^3^ Department of Surgery Seoul National University College of Medicine Seoul South Korea; ^4^ Department of Surgery Seoul National University Hospital Seoul South Korea; ^5^ Ischemic/Hypoxic Disease Institute Seoul National University Medical Research Center Seoul South Korea

**Keywords:** drug screening, Hepatitis B virus, hepatocellular carcinoma, integrative characterization, patient‐derived organoids

## Abstract

**Background:**

Hepatocellular carcinoma (HCC) is one of the most aggressive malignancies and is characterized by a high degree of heterogeneity. However, HCC treatment remains difficult, highlighting the need to better define molecular subgroups of HCC and understand their potential relationships with therapeutic response.

**Methods:**

In this study, we established 20 patient‐derived organoids and 12 patient‐derived cell lines from 20 individuals diagnosed with HCC. These models were comprehensively characterized using whole‐exome sequencing, RNA sequencing, drug screening, and hepatitis B virus (HBV)‐targeting PCR with a focus on HBV‐related HCC.

**Results:**

Based on transcriptomic profiling, this study classified HCC into two molecular subtypes, aligning with previously reported classifications. Drug screening revealed that the greater enrichment of the PI3K/AKT/mTOR signalling pathway was associated with increased sensitivity to everolimus and lenvatinib. Integrated transcriptomic and drug‐response analyses further identified candidate genes associated with lenvatinib response. In addition, polymerase chain reaction (PCR) analysis targeting the HBV surface, core, and X genes demonstrated noticeable discrepancies relative to the clinically reported HBV infection status. Notably, HBV DNA detection was associated with activation of the calcium signalling pathway and oxaliplatin response.

**Conclusions:**

This study provides a valuable resource for investigating the biology and pharmacological characteristics of HCC, particularly in the context of molecular subtypes and HBV‐associated mechanisms. Ultimately, these findings offer additional insights into HCC heterogeneity and drug response and may serve as a basis for further investigation of molecular determinants of therapeutic response.

## Highlights

1. 20 HCC‐derived organoids, three adjacent non‐tumour tissue‐derived organoids, and 12 HCC‐derived cell lines from 20 patients were established and biobanked.
2. Integrative analyses combining drug screening, whole‐exome sequencing, and RNA sequencing revealed molecular subtypes and identified candidate determinants of lenvatinib response.
3. HBV‐associated analyses revealed potential relationships linking HBV status, calcium signalling, and oxaliplatin response.

## Graphical abstract

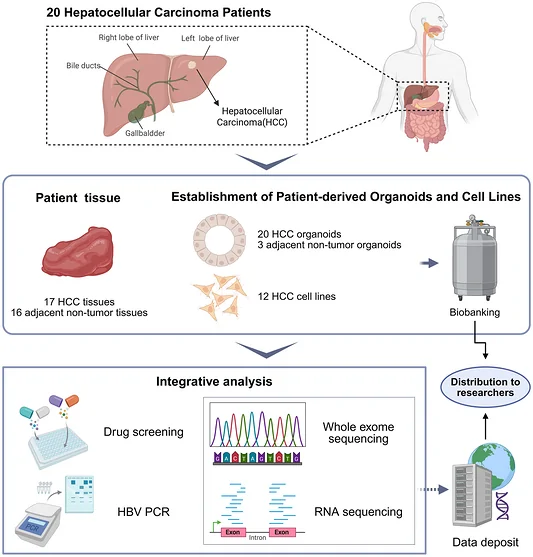

## 1 BACKGROUND

Hepatocellular carcinoma (HCC) is the sixth most common cancer worldwide and the 3rd leading cause of cancer death.^1^ It is the most common form of primary liver cancer, which accounts for 75% to 85% of all cases.^2^ One of the main causes of HCC is infection with the Hepatitis B Virus (HBV), which was responsible for over 50% of global cases in 1990.^3^ Although this proportion declined to 42% by 2019, the burden remains especially high in East Asia, where approximately 62% of HCC cases are attributed to HBV infection.^3^, ^4^ To enhance the understanding of the molecular mechanisms underlying HCC, we generated 20 HCC‐derived organoids and 3 adjacent non‐tumour tissue‐derived organoids, as well as 12 HCC‐derived cell lines from 20 Korean patients at Seoul National University Hospital. Using matched primary tumour tissues, these models underwent integrative molecular and pharmacological profiling, including whole‐exome sequencing, RNA sequencing, and drug screening. Additionally, HBV PCR targeting the HBV surface, core, and X genes was conducted to investigate HBV‐associated molecular features.

HCC poses significant treatment challenges due to its highly heterogeneous nature, limited therapeutic options, and variable responses to chemotherapeutic agents. Therefore, it is crucial to identify molecularly defined subgroups of HCC and develop tailored therapeutic strategies for each subgroup. Patient‐derived organoids provide a relevant preclinical platform for capturing tumour heterogeneity and evaluating drug responses in a patient‐specific context. Here, we classified established HCC organoids into two distinct molecular subtypes and validated this classification using the TCGA‐LIHC dataset. Furthermore, drug‐response profiling demonstrated significant differences between the two subgroups, suggesting the molecular subgrouping may be relevant to understanding differential therapeutic responses in HCC.

HBV infection promotes hepatocarcinogenesis through chronic inflammation, progressive cirrhosis, and direct viral genome integration. While clinical HBV status is typically assessed using serological markers (e.g., HBsAg, HBeAg, and their corresponding antibodies) and by measuring HBV DNA levels in the blood, these tests do not detect the presence of HBV DNA integration or covalently closed circular DNA (cccDNA).^5^, ^6^ This distinction is important: integrated HBV DNA fragments can induce genomic instability and insertional activation of oncogenes, while cccDNA sustains persistent viral transcription.^7^ Thus, clinical HBV markers and PCR‐based HBV DNA detection may provide complementary information reflecting different biological layers of HBV infection. To further characterize intra‐tumoral HBV DNA status, we conducted a PCR analysis targeting the HBV surface, core, and X genes in HCC samples and revealed discrepancies between HBV PCR results and clinical information. Furthermore, when integrated with transcriptomic analysis and drug screening outcomes, these findings suggested an association between HBV DNA status and drug response.

Overall, this study offers an integrative analysis of genomic, transcriptomic, pharmacological, and HBV‐associated features in patient‐derived HCC models. By establishing HCC‐derived models and molecular subgroups, identifying drug‐associated genes, characterizing HBV‐related signalling alterations, and examining their associations with therapeutic vulnerabilities, our findings yield novel insights into HCC biology and highlight opportunities for developing more precise and effective treatment approaches.

## 2 METHODS

### 2.1 Establishment of human HCC and adjacent non‐tumour tissue‐derived organoids

Fresh tumour tissues were rinsed in a basal culture medium (Dulbecco's modified Eagle medium/F12 supplemented with 1% penicillin/streptomycin, 1x Glutamax and 10% fetal bovine serum). The tissues were finely minced using sterilized scissors and then enzymatically digested in a rotating incubator at 37°C for 90 min. Following tissue dissociation, the suspension was strained through a 100 µM cell strainer to eliminate residual large fragments. The filtered cells were centrifuged at 1500 rpm for 3 min. To remove erythrocytes, the cell pellet underwent red blood cell lysis buffer for 10 min, and the reaction was neutralized with bovine calf serum and medium, followed by another centrifugation at 1500 rpm for 3 min. The recovered cells were mixed with BME and dispensed into T‐25 flasks. Once the BME had polymerized, either HCC organoid medium (basal culture medium with 50% L‐WRN conditioned medium, 50 ng/mL human EGF, 100 ng/mL human FGF‐10, 25 ng/mL human HGF, 10 µM Forskolin, 1x B27, 10 mM Nicotinamide, 500 nM A83‐01, 1.25 mM n‐Acetyl cysteine, 10 µM Rho inhibitor Y‐27632, 3 µM CHIR‐99021, 20 ng/mL TGF‐alpha, 10 nM Gastrin and 100 µg/mL Primocin) or adjacent non‐tumour tissue‐derived organoid medium (advanced Dulbecco's modified Eagle medium/F12 with 10% R‐spondin 1 conditioned medium, 50 ng/mL human EGF, 100 ng/mL human FGF‐10, 25 ng/mL human HGF, 10 µM forskolin, 1x B27, 10 mM Nicotinamide, 500 nM A83‐01, 1 mM n‐Acetyl cysteine, 10 nM Gastrin, 10 mM HEPES, 500 pM Wnt‐surrogate‐Fc, 100 ng/mL Noggin, 1x B27 minus vitamin A, 10 µM SB202190 and 100 µg/mL Primocin) was added to the flask, depending on the tissue origin. Cultures were incubated at 37°C under humidified conditions with 5% CO_2_.

### 2.2 Establishment of human HCC cell line‐derived organoids

The HCC cell line‐derived organoid, designated SNU‐0000‐CO, was established from a previously cultured HCC cell line. After removing the culture medium, the cells were enzymatically detached from a T‐75 flask using trypsin. The cell suspension was neutralized with bovine calf serum and medium, then centrifuged at 1500 rpm for 3 min. Cells collected in the pellet were mixed with BME and subsequently dispensed into T‐25 flasks. After the gel polymerized, HCC organoid medium was added, and the cultures were maintained at 37°C in a humidified atmosphere containing 5% CO_2_. Established organoid samples were deposited at the Korean Cell Line Bank for global distribution.

### 2.3 Organoid culture

For passaging, the culture media was first removed, and the organoids embedded in BME gel were dissociated using TrypLE Express. Digestion was carried out at 37°C for approximately 10 min before proceeding to the next processing step. To neutralize the reaction, bovine calf serum and medium were added, followed by centrifugation at 1500 rpm for 3 min to collect the cells. The collected cells were embedded in BME gel and dispensed as 60–70 µL droplets onto T‐25 flasks. After the gel solidified, liver organoid medium was introduced into the cultures. Medium replacement was subsequently performed every three days.

### 2.4 Establishment of human HCC‐derived cell lines and HCC organoid‐derived cell lines

Tumour cells isolated from human HCC samples were suspended in Opti‐MEM 1 and plated into T‐25 or T‐75 flasks. When stromal cells proliferated during the initial culture phase, tumour cells were selectively enriched using either localized trypsinization or mechanical scraping. Following establishment of the primary cultures, the cells were expanded in R10 medium (RPMI 1640 with 10% FBS and 1% penicillin/streptomycin). Cells were maintained at 37°C with 5% CO_2_.

HCC organoid‐derived cell lines, designated as SNU‐0000T, were established from previously cultured HCC organoids. After removing the culture medium, the organoids were enzymatically dissociated using TrypLE Express by pipetting, followed by incubation at 37°C for approximately 10 min. Following enzymatic digestion, the cells were mixed with bovine calf serum and medium, then centrifuged at 1500 rpm for 3 min. The resulting dissociated cells were transferred to T‐25 or T‐75 flasks and then cultured in the culture medium (R10 medium with liver organoid medium in a 1:1 ratio) under standard humidified incubation conditions at 37°C with 5% CO_2_. Established cell line samples were deposited in the Korean Cell Line Bank for global distribution.

### 2.5 Genomic DNA extraction and DNA fingerprinting analysis

For genomic DNA isolation, organoids and cell lines were processed using the QIAamp DNA Mini Kit, while tissue samples were processed with the QIAamp Fast DNA Tissue Kit. The extracted genomic DNA underwent amplification using the AmpFISTR identifier polymerase chain reaction (PCR) amplification kit. This PCR process, performed in a single cycle, targeted 26 short tandem repeat loci. These loci comprise highly polymorphic microsatellite regions, enabling precise DNA fingerprinting analysis. The STR profiling results are summarized in Table S1.

### 2.6 Whole exome sequencing analysis

All samples underwent whole‐exome capture using one of the following kits: SureSelect Human All Exon V5+UTR‐post, V5‐post, or V6‐post kit (Agilent Technologies). Sequencing was conducted on the HiSeq 2500 (Illumina). For organoid and cell line samples, the 100 bp paired‐end reads option was utilized, while tissue samples were sequenced with the 200 bp paired‐end reads option. Sequencing data were analyzed through an internally developed bioinformatics workflow. Initially, paired‐end reads were mapped against the UCSC hg19 human reference genome (equivalent to GRCh37 from NCBI, Feb. 2009) using the mapping program BWA (version 0.7.12), generating BAM‐format alignment files with BWA‐MEM. Picard‐tools (version 1.130) was subsequently used to identify and remove PCR duplicate reads. Local realignment of reads within the BAM files was performed to adjust sequences mapped to the same start position, utilizing MarkDuplicates.jar, which required sorted input reads. Subsequently, the Genome Analysis Toolkit (GATK) was used to recalibrate base quality scores and refine alignments in regions surrounding indels. For each sample, variant genotyping was carried out using the HaplotypeCaller of GATK (GATK v3.4.0), which identified potential single nucleotide polymorphisms and small indels based on the pre‐processed BAM files. Somatic mutation detection was conducted with MuTect2 (involved in GATK v3.8.0), operating in tumour‐normal mode with default parameters. This step used both the reference genome and the aligned sequence data from tumour samples. The resulting variant calls were annotated by SnpEff v4.1 g and exported in VCF format. Initial filtering of variants was performed using dbSNP version 142 and single nucleotide polymorphism data from the 1000 Genomes Project. Further annotation and filtering with SnpEff were conducted using additional databases, including dbNSFP 2.9, ClinVar, and ESP6500.

### 2.7 RNA‐sequencing analysis

Cell lysates were subjected to total RNA extraction using TRIzol and the Qiagen RNeasy kit. Complementary DNA (cDNA) libraries were constructed and sequenced on the NovaSeq6000 platform to generate paired‐end reads. FastQC (version 0.11.7) was initially applied to assess the quality of the generated sequencing data. Adapter sequences and low‐quality bases were eliminated from the raw reads with Trimmomatic (version 0.38). The trimmed reads were then mapped against the UCSC hg19 human reference genome using HISAT2 (version 2.1.0), which is based on the Bowtie2 aligner (version 2.3.4.1). Subsequent transcriptome reconstruction, including the characterization of newly identified transcripts, annotated transcripts, and alternatively spliced transcripts, was carried out using StringTie (version 2.1.3b).

Batch effects associated with tissue storage method (RNAlater and isopentane) were corrected using the ComBat_seq function implemented in the sva R package (version 3.50.0).

### 2.8 FFPE block production and haematoxylin and eosin staining

Organoids embedded within the gel dome were gently detached from the bottom of the culture flask using a pipette tip with the end trimmed. The harvested gel material was transferred into a 1.5 mL tube filled with phosphate‐buffered saline. After a brief centrifugation, the supernatant was carefully removed multiple times to minimize residual gel, leaving mostly organoids. The resulting cell pellet was suspended in 2% agarose gel and allowed to polymerize. The agarose‐embedded blocks were fixed with 10% paraformaldehyde at room temperature for 30 min. After fixation, the blocks were cut into 4 µM‐thick slices and subjected to routine haematoxylin and eosin staining.

### 2.9 Drug sensitivity test for 3D organoids

Fragmented organoids (2K–10K per well) were seeded along the edges of wells in 96‐well white plates using a 7:3 mixture of HCC organoid medium and BME gel. The gel mixture was allowed to polymerize for 15 min at 37°C in a 5% CO_2_ incubator. Each well was then supplemented with 20 µL of prewarmed HCC organoid medium. Following 96 h of incubation, each well received 20 µL of drug solution prepared by serial dilution. Following an additional 72 h treatment period, the Cell‐Titer‐Glo assay was used to quantify cell viability, and luminescence was measured using a Multiskan Ascent Microplate Luminometer. Measurements were conducted in three technical replicates to assess assay reproducibility.

Drug concentrations were selected based on previously reported drug sensitivity data. A pilot dose‐response study was performed to determine the concentration range and dilution factor, with concentrations selected to encompass the expected IC50. Area under the curve (AUC) values were calculated using the trapezoidal rule over the tested concentration range, with drug concentrations represented on a log10 scale and cell viability normalized to the negative control. IC50 values were derived from the fitted curves using the minpack.lm R package (version 1.2‐4).

### 2.10 siRNA transfection

For gene knockdown experiments, fragmented organoids (SNU‐7238‐TO, SNU‐7442‐TO, and SNU‐9204‐TO) were transfected with siRNA. A negative control siRNA and siRNAs targeting SERPINA5, VTN, C3, and F11 (Bioneer) were introduced into the cells at a final concentration of 30 nM using Lipofectamine RNAiMAX (Invitrogen), in accordance with the manufacturer's protocol. After 6 h of incubation, the transfected organoids were seeded into culture flasks, and liver organoid medium was added. Antibiotics were omitted from the medium under all conditions, as recommended by the manufacturer, to maintain cell viability during the transfection process. The organoids were incubated for 72 h before assessment of transfection efficiency.

To validate knockdown efficiency, total RNA was isolated with TRIzol and the Qiagen RNeasy kit, as described above, and subsequently converted to cDNA using the Qiagen reverse transcription kit. Real‐time qPCR was then carried out with the Bioneer master mix to assess knockdown efficiency. Triplicate measurements were obtained for each sample, and relative transcript abundance was determined using the 2^−ΔΔCt^ method, with GAPDH as the housekeeping gene for normalization.

For the drug sensitivity test, the organoids were seeded into 96‐well white plates as described previously. Once the gel had polymerized, each well was first supplemented with 20 µL of prewarmed HCC organoid medium, followed by 20 µL of drug solution prepared by serial dilution. After 72 h, cell viability was assessed as mentioned above.

### 2.11 HBV PCR

For HBV core gene PCR screening, 1 µL of genomic DNA from each sample was used as a template in a 14 µL PCR reaction mixture. For PCR amplification, the reaction mixture was prepared with 1.5 µL of 10× PCR buffer without MgCl_2_, 1 µL of MgCl_2_, 0.5 µL of dNTPs, 0.25 µL each of the forward and reverse primers, and 0.08 µL of Taq DNA polymerase (Intron Biotechnology). PCR amplification was performed using the GeneAmp PCR System 9700 (Applied Biosystems), with 35 cycles of denaturation at 94°C, annealing at 68°C, and extension at 72°C. SNU‐3058, an HBV‐positive sample, served as a positive control, while diluted water was used as a negative control. To confirm the presence and integrity of human genomic DNA, the COX1 gene was amplified as an internal control. In addition to the HBV core gene, the HBV surface gene, the HBV X gene, and the COX1 gene were also amplified using distinct primer sets under the same PCR conditions, with the exception of the annealing temperatures and extension time. The detailed PCR conditions and primer sequences are summarized in Table S2.

### 2.12 Statistical analysis

All statistical analyses and data visualizations were performed using R program version 4.3.1 (R Foundation for Statistical Computing), GraphPad Prism (version 5.00), Cytoscape (version 3.10.3), and GSEA (version 4.3.3). A range of statistical tests was applied to analyze the clinical, transcriptomic, and experimental datasets. These included, but were not limited to, Spearman's correlation coefficient test, chi‐squared test, Fisher's exact test, Wilcoxon rank‐sum test, and log‐rank test.

For transcriptomic analysis, the normalization and transformation methods were selected according to the specific downstream analysis. Variance‐stabilizing transformation was applied using the vst function in the DESeq2 R package (version 1.42.1) for principal component analysis (PCA) and consensus clustering. DESeq2 normalization was performed using the estimateSizeFactors function in DESeq2, and the resulting normalized expression values were used for Gene Set Enrichment Analysis (GSEA). Regularized log transformation was performed using the rlog function in DESeq2 for gene set variation analysis (GSVA). Read counts were used as input for differential expression analysis and for calculating Spearman correlation coefficients between paired samples. FPKM values were used for Spearman correlation analyses between gene expression levels and drug‐response AUC values.

To compare gene expression between two groups, differential expression analysis was conducted using DESeq2, which computed log2 fold changes and corresponding *p*‐values for each gene. Cluster determination was carried out using the ConsensusClusterPlus method R package (version 1.51.1). Consensus clustering was performed with 1,000 resampling iterations, in which 80% of samples were randomly selected in each iteration, using all features and hierarchical clustering with a Spearman correlation‐based distance. Liver cancer‐specific molecular classification was assessed using the MS.liverK package (version 1.0.0). Gene set enrichment scores at the individual level were estimated with the GSVA package (version 1.50.5), whereas gene ontology (GO) enrichment analysis was assessed using the clusterProfiler package (version 4.10.1). Survival analyses were conducted with the survival package (version 3.8‐3).

## 3 RESULTS

### 3.1 Establishment of human HCC‐derived organoids and cell lines

We established organoids and cell lines from 20 HCC patients who underwent surgical resection at Seoul National University Hospital. Along with paired tissue, these were subjected to integrative genomic, transcriptomic, drug response, and HBV PCR analyses. In total, the dataset comprised 20 HCC organoids, 3 organoids from adjacent non‐tumour tissues, 12 HCC cell lines, 17 paired HCC tissues, and 18 adjacent non‐tumour tissues (Table S3).

The established HCC organoids primarily exhibited two types of morphological characteristics: budding, lumen‐forming shapes (e.g., SNU‐7490‐TO, SNU‐7941‐TO, and SNU‐8090‐TO) and round shapes, with the majority forming solid, compact structures without a lumen (e.g., SNU‐6978‐CO, SNU‐7012‐TO, and SNU‐7238‐TO) (Figure 1A). These morphologies were the most common and have been reported as representative features of HCC organoids.^8^, ^9^ In contrast, organoids derived from adjacent non‐tumour tissues displayed cystic forms (Figure 1B), consistent with the typical characteristics of normal liver organoids.^9^ Notably, haematoxylin and eosin staining demonstrated that morphologies of tumour organoids reflected those of their paired tissues (Figure 1C; Figure S1A). By comparison, the established HCC cell lines exhibited distinct morphological phenotypes, including polygonal, fibroblast‐like, and spindle‐like shapes (Figure S1B).

**FIGURE 1:**
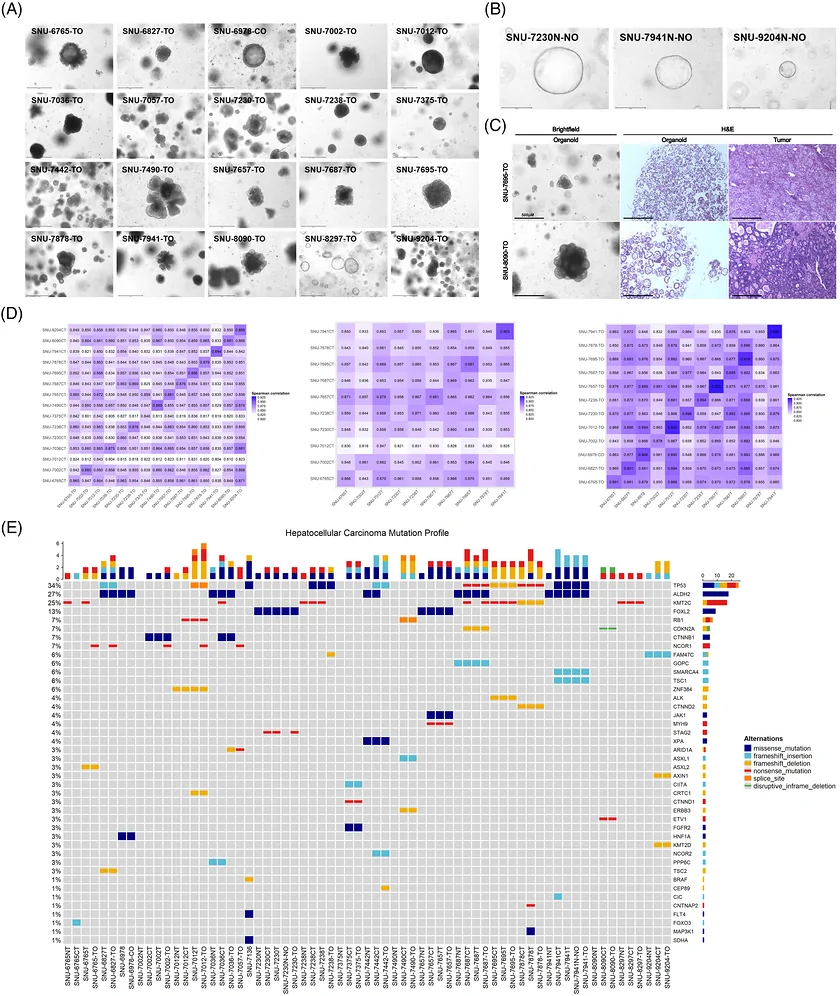
Establishment of HCC‐derived organoids and cell lines and comparison with paired tissues. (A) Representative images of 20 HCC‐derived organoids (scale bars, 500 µm). (B) Representative images of three adjacent non‐tumour tissue‐derived organoids (scale bars, 500 µm). (C) Representative bright‐field microscopy images and haematoxylin and eosin staining of HCC‐derived organoids and paired tissues from the patients SNU‐7695 and SNU‐8090 (scale bars, 500 µm). (D) Heatmaps showing the Spearman's correlation coefficients of the mRNA expression (left) between HCC‐derived organoids and paired tissues, (middle) between HCC‐derived cell lines and paired tissues, and (right) between HCC‐derived cell lines and organoids. (E) Mutational profile of patient‐derived samples and paired tissues (*n* = 21 pairs). Enrichment is shown as the normalized enrichment score (NES), nominal *p*‐value, and FDR *q*‐value.

### 3.2 Comparison of organoids and cell lines with paired tissues

To assess transcriptomic similarities, we calculated Spearman's correlation coefficients among organoids, cell lines, and their paired tissues (Figure 1D). Tumour organoids more faithfully recapitulated the transcriptomic profiles of their corresponding tissues compared with cell lines. However, when examining overall patterns, tumour organoids and cell lines exhibited greater similarity to each other than to other sample types. Within tumour samples, each case displayed distinct expression patterns, whereas normal samples showed relatively homogeneous transcriptomic profiles across cases (Figure S1C,D). Furthermore, established normal organoids did not retain sample‐specific expression signatures from their matched normal tissues (Figure S1E).

The mutational profile showed the overall mutations across all established samples and origin tissues (Figure 1E). We collected 42 cancer driver genes from Cancer Gene Census in COSMIC, the Catalogue of Somatic Mutations in Cancer, and GDC, the Genomic Data Commons data portal. The mutational landscape of our established samples was largely consistent with that of their matched origin tissues. TP53, one of the most frequently mutated genes in HCC, was altered in 35.6% of the TCGA‐LIHC cohort, 35.3% of SNU‐HCC tissues, and 42.1% of SNU‐HCC organoids. In addition, mutations in CTNNB1, CDKN2A, and RB1—genes commonly reported in HCC—were detected at frequencies comparable to those in previous studies,^10^ with 11.8% in SNU‐HCC tissues and 10.5% in SNU‐HCC organoids. These findings indicate that our dataset faithfully reflects the mutational spectrum of HCC observed in public cohorts, supporting the reliability of our results.

Comparative analysis of the mutational profile revealed that a subset of genetic alterations was shared between tumour and paired normal samples, suggesting the presence of background variants. In contrast, distinct mutations of well‐established tumour suppressor genes such as TP53, CDKN2A, and RB1 were observed exclusively in tumour samples. These tumour‐specific alterations underscore their potential role as key oncogenic factors contributing to initiation and advancement of HCC.

### 3.3 Transcriptomic analysis identifies two molecular subtypes of HCC organoids

We performed unsupervised consensus clustering on our 18 established organoids (excluding SNU‐7941‐TO) based on mRNA expression, selecting the top 2000 most variable genes. This analysis identified two distinct molecular subtypes, designated as C1 and C2 (Figure 2A; Figure S2A–C), which were further confirmed by PCA and a gene‐expression heatmap (Figure 2B). Additionally, we validated the clustering by comparing it with clusters from previous studies (Figure 2C).^10^, ^11^, ^12^, ^13^ Clustering of our transcriptomic data using the R package MS.liverK^14^ and signature genes identified in previous reports (Table S4) reproduced comparable two‐subgroup distributions (Figure S3A–D; Table S5). Validation showed that C1 was characterized by well‐differentiated features with favourable survival, whereas C2 exhibited proliferative and metastatic characteristics associated with poor survival.

**FIGURE 2:**
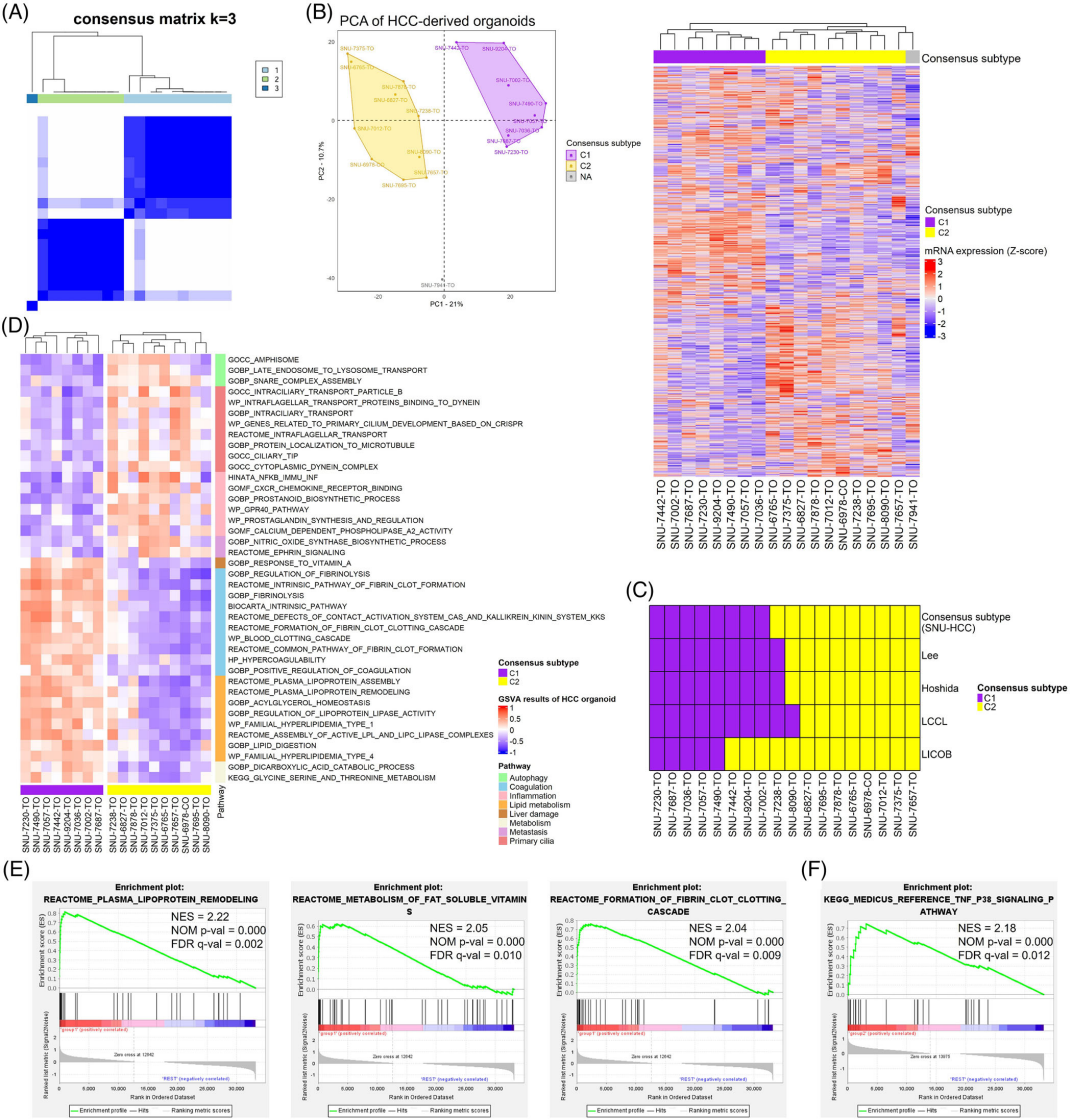
Molecular subtypes of HCC‐derived organoids. (A) Consensus clustering (*k* = 3) based on mRNA expression identified two major molecular subtypes (C1 and C2) among 18 HCC‐derived organoids; SNU‐7941‐TO was not assigned to either subtype. (B) (left) PCA and (right) heatmap of the transcriptomic profile for HCC‐derived organoids (*n* = 19, including SNU‐7941‐TO). The heatmap shows mRNA expression levels as Z‐scores, with red and blue indicating higher and lower relative expression, respectively. (C) Comparison of subtyping from SNU‐HCC organoids (*n* = 18) with the subtyping from previously reported signature genes. (D) GSVA showing distinct enrichment patterns between C1 and C2 subtypes (*n* = 18). Rows represent enriched pathways, and columns represent individual organoids, with the annotation bar indicating the corresponding C1/C2 subtype. Colours indicate GSVA scores, with positive and negative values representing relatively higher and lower pathway activity, respectively. (E, F) GSEA plots showing enriched pathways in (E) C1 (*n* = 8) and (F) C2 (*n* = 10). Enrichment is shown as the NES, nominal *p*‐value, and FDR *q*‐value. NES, normalized enrichment score.

We performed GSVA to identify the specific enriched signalling pathways for the C1/C2 subtypes (Figure 2D). C1 was associated with increases in coagulation, lipid metabolism, metabolism, and liver damage‐related pathways, whereas C2 was enriched in pathways related to primary cilia, inflammation, autophagy, and metastasis. Additionally, GSEA revealed distinct pathway activations in each subtype, indicating that lipid metabolism and coagulation‐related pathways were enriched in C1 (Figure 2E), while an inflammation‐related pathway was enriched in C2 (Figure 2F).

The mutation frequencies for the C1/C2 subtypes were consistent with previous reports. TP53 and CTNNB1 were the most common driver mutations in HCC, with TP53 mutations typically associated with high stemness, poor prognosis, and proliferative subtypes,^12^, ^15^, ^16^ and CTNNB1 mutations enriched in subtypes exhibiting low stemness, favourable prognosis, and metabolomic reprogramming.^12^, ^15^, ^16^, ^17^, ^18^ Although statistical significance was limited by the small number of organoids, these mutational patterns were reflected in our organoid models (Figure S4): CTNNB1 mutations appeared exclusively in C1, while TP53 mutations were more prevalent in C2.

Overall, the features of the C1/C2 subtypes were consistent with previous studies, supporting the reproducibility of these molecular features in our HCC organoid models. Consequently, the pathways associated with the C1/C2 subtypes may provide additional insights into their biological characteristics.

### 3.4 Verification of the C1/C2 subtype classification in the TCGA‐LIHC cohort

We extracted 92 signature genes associated with enriched pathways in the C1/C2 subtypes (Figure 3A). Using these genes, we performed unsupervised consensus clustering on the TCGA‐LIHC dataset (*n* = 361), identifying two distinct clusters (Figure 3B). The TCGA‐LIHC Kaplan–Meier curve revealed that C2 had a significantly poorer prognosis than C1 (*p* = .027, hazard ratio = 1.638, 95% confidence interval [CI]: 1.05–2.55), as previously described (Figure 3C).^11^, ^13^

**FIGURE 3:**
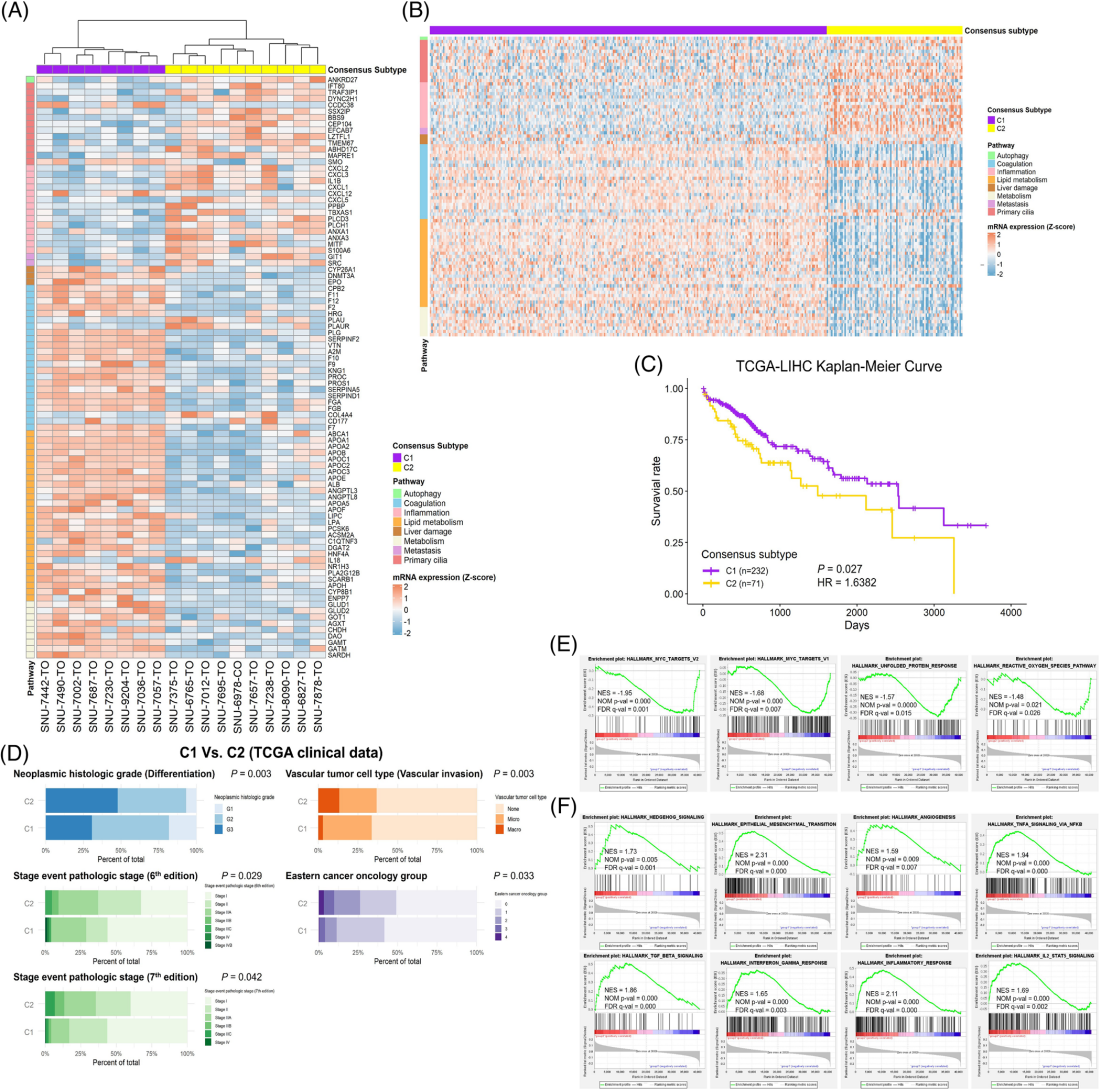
Cross‐validation of the C1/C2 subtypes in the TCGA‐LIHC cohort. (A, B) Heatmap showing expression patterns of 92 signature genes in (A) SNU‐HCC organoids (*n* = 18) and (B) TCGA‐LIHC cohort (*n* = 361). Rows represent the 92 signature genes, and columns represent individual samples. The coloured annotation bars indicate the corresponding C1/C2 subtype and the functional pathway assigned to each signature gene. The heatmap shows mRNA expression levels of the 92 signature genes as Z‐scores, with red and blue indicating higher and lower relative expression, respectively. (C) Kaplan–Meier curve showing the survival of the following two subtypes of the TCGA‐LIHC dataset: C1 (*n* = 232) and C2 (*n* = 71). The *p*‐value was calculated by the log‐rank test. HR, hazard ratio. (D) Comparison of clinicopathological characteristics of C1/C2 subtypes in the TCGA‐LIHC cohort. Horizontal stacked bar plots show the percentage distribution of samples within each clinical category. *p*‐values were calculated by the chi‐squared test for neoplasmic histologic grade (differentiation) (*n* = 345) and vascular tumour cell type (vascular invasion) (*n* = 305), and by Fisher's exact test for stage event pathologic stage (*n* = 116, 6th edition; *n* = 217, 7th edition) and eastern cancer oncology group (*n* = 282). (E, F) GSEA plot showing enriched pathways in (E) C1 (*n* = 269) and (F) C2 (*n* = 92). Enrichment is shown as the NES, nominal *p*‐value, and FDR *q*‐value.

Furthermore, we examined the clinical features of the C1/C2 subtypes within the TCGA‐LIHC dataset (Figure 3D). Regarding neoplasmic histologic grade, C1 exhibited a tendency toward a well‐differentiated histologic grade compared with C2, which accounted for a higher proportion of higher‐grade tumours (*p* = .003, Cramer's V = .17). Analysis of vascular tumour cell type, an indicator of vascular invasion, revealed that C2 had a more advanced profile, with a higher frequency of macrovascular invasion than C1 (*p* = .003, Cramer's V = .18). Both the 6th and 7th editions of stage event pathologic stage showed that C2 had a higher percentage of late‐stage tumours compared with C1 (*p* = .029, Cramer's V = .23, 6th edition; *p* = .042, Cramer's V = .16, 7th edition). Additionally, the Eastern Cancer Oncology Group (ECOG) performance status was significantly higher in C2, indicating a worse overall patient status (*p* = .033, Cramer's V = .15).

GSEA analysis of the TCGA‐LIHC dataset was performed to identify the enriched hallmark pathways for the C1/C2 subtypes. The Myc target pathways, unfolded protein response, and reactive oxygen species pathway were enriched in C1 (Figure 3E). The Myc pathway is known to be related to metabolism,^19^ consistent with the feature of C1. Additionally, the enrichment of the unfolded protein response and reactive oxygen species pathway may result from the activation of metabolic pathways. On the other hand, C2 was predominantly enriched in pathways related to hedgehog signalling, metastasis, and inflammation (Figure 3F). Hedgehog signalling is deeply related to primary cilia,^20^, ^21^ and plays a vital role in proliferation and invasiveness in HCC.^22^, ^23^ Epithelial‐mesenchymal transition, angiogenesis, and TGF‐beta signalling are associated with metastasis, while TNF‐alpha signalling via NFKB, interferon‐gamma response, inflammatory response, and IL2‐STAT5 signalling are related to inflammation.

In summary, cross‐validation of the TCGA‐LIHC dataset demonstrated that C1 was characterized by clinical features of well‐differentiated tumours with a milder prognosis and was enriched in metabolic pathways. Conversely, C2 was associated with features of late‐stage, invasive tumours and a poor prognosis, enriched in proliferation, metastasis, and inflammation‐related pathways. These results align with previous reports, supporting the reproducibility of the molecular features captured by 92 signature genes. Notably, several studies have shown that a subtype enriched with metastasis and immune‐related pathways is associated with poor prognosis, whereas another subtype enriched with metabolism‐related pathways is associated with a milder prognosis.^16^, ^17^, ^18^

### 3.5 HCC organoids exhibit heterogeneous responses to drug treatment

We performed drug screening using 18 established HCC organoids treated with 8 different drugs (Figure 4A; Figure S5; Tables S6 and S7). Sorafenib, lenvatinib, and regorafenib are the clinically established first‐ and second‐line systemic therapies for advanced HCC. To complement these targeted agents, we included doxorubicin, oxaliplatin, and 5‐FU, which are key chemotherapeutic agents used in TACE, HAIC, and FOLFOX protocols. Tacrolimus and everolimus represent essential immunosuppressive therapies routinely administered after liver transplantation. Together, this panel encompasses the major therapeutic classes in contemporary HCC management.

**FIGURE 4:**
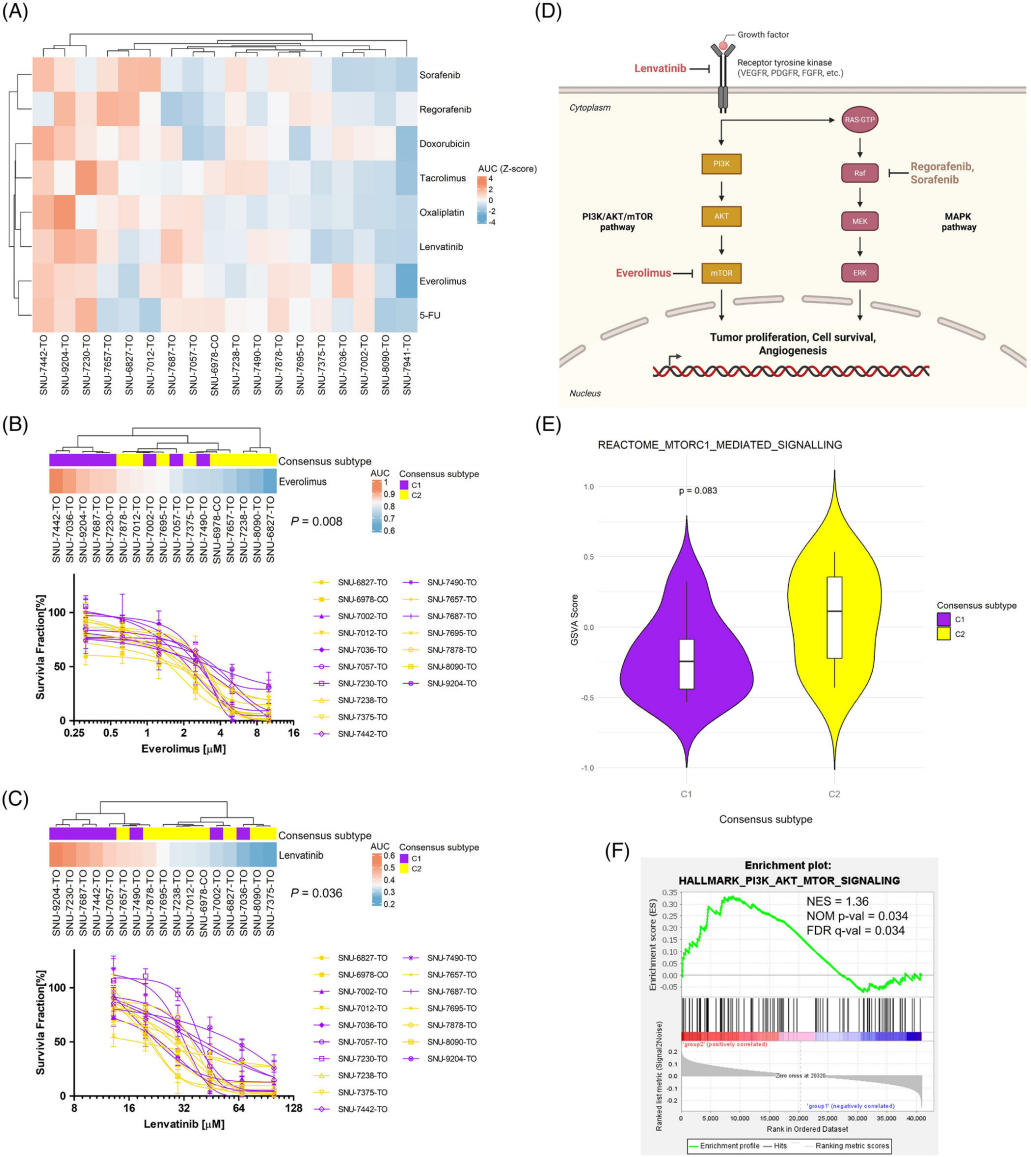
Heterogeneous drug response of HCC organoids. (A) Heatmap showing drug response for HCC organoids (*n* = 18) against eight chemotherapeutic agents, which include sorafenib, regorafenib, doxorubicin, tacrolimus, oxaliplatin, lenvatinib, everolimus, and 5‐FU. All experiments were performed in technical triplicate, and the mean AUC values were used for subsequent analyses. Drug sensitivity is represented by AUC Z‐scores, with red and blue indicating higher and lower AUC Z‐scores, respectively. (B, C) Top, heatmap showing drug response of (B) everolimus and (C) lenvatinib, with the colour annotation representing the C1/C2 subtypes. The colour scales represent AUC values, with the ranges indicated by the corresponding colour bars. *p*‐values indicate comparisons of AUC values between C1 and C2 subtypes and were calculated by the Wilcoxon rank‐sum test. Bottom, drug‐response curves of (B) everolimus and (C) lenvatinib (*n* = 17). Data are presented as mean ± SD. (D) Mechanism of action of receptor tyrosine kinase signalling‐related drugs. (E) Violin plot representing GSVA enrichment scores of the REACTOME_MTORC1_MEDIATED_SIGNALLING pathway by the C1/C2 subtypes. The number of organoids analyzed was *n* = 18 (*n* = 8, C1; *n* = 10, C2). *p*‐value was calculated by the Wilcoxon rank‐sum test. (F) GSEA plot showing enrichment of the HALLMARK_PI3K_AKT_MTOR_SIGNALING gene set in C2 of the TCGA‐LIHC dataset (*n* = 269, C1; *n* = 92, C2). Enrichment is shown as the NES, nominal *p*‐value, and FDR q‐value. NES, normalized enrichment score. SD, standard deviation.

Among the drugs tested, the AUC values for everolimus and lenvatinib differed significantly between the C1 and C2 subtypes (*p* = .008, Cliff's delta = .75, 95% CI: .39–.91, everolimus; *p* = .036, Cliff's delta = .61, 95% CI: .14–.86, lenvatinib) (Figure 4B,C). Referring to the mechanisms of action of receptor tyrosine kinase (RTK) signalling‐associated drugs (Figure 4D), both lenvatinib, a multitarget tyrosine kinase inhibitor, and everolimus, an mTOR inhibitor, exhibited higher sensitivity in C2 compared with C1. In contrast, MAPK pathway‐associated drugs such as regorafenib and sorafenib, both targeting Raf, showed no significant AUC differences (Figure S6). These findings suggest a potential difference in PI3K/AKT/mTOR pathway activity between C1 and C2. Consistently, GSVA revealed a higher enrichment score of mTORC1‐mediated signalling in C2 (*p* = .083, Cliff's delta = −.50, 95% CI: −.80 to .00) (Figure 4E). Cross‐validation using the TCGA‐LIHC dataset further confirmed that the PI3K/AKT/mTOR pathway was enriched in C2 (Figure 4F).

Comprehensively, C2, enriched with the PI3K/AKT/mTOR pathway, exhibited greater sensitivity to everolimus and lenvatinib, which target mTOR and RTKs, respectively. Supporting this observation, Ji et al.^13^ reported that the mTOR‐high HCC subgroup was particularly sensitive to PI3K/AKT/mTOR inhibitors. Similarly, Chen et al.^24^ demonstrated that HCC patients with high expression of KMD6A, an activator of the PI3K/AKT/mTOR cascade, exhibited increased sensitivity to lenvatinib. In triple‐negative breast cancer, Huang et al.^25^ further found that DRPA1, which promotes proliferation, invasion, and migration both in vitro and in vivo through the activation of mTOR signalling, enhanced sensitivity to the mTORC1 inhibitor everolimus in both in vitro and in vivo models. Collectively, these findings suggest an association between PI3K/AKT/mTOR pathway enrichment and the differential sensitivity of C1 and C2 to everolimus and lenvatinib.

### 3.6 Integrated drug screening and transcriptomic analysis indicate the lenvatinib‐associated signalling pathway

Lenvatinib is one of the most commonly used chemotherapeutic drugs for first‐line treatment of HCC. However, HCC is a highly heterogeneous malignancy and exhibits variable response rates to lenvatinib. Therefore, identifying predictive factors for lenvatinib efficacy is critical for optimizing therapeutic outcomes. To this end, we calculated Spearman's correlation coefficients between gene expression levels and drug sensitivity (Figure 5A). A total of 415 genes showed significant correlations with lenvatinib response (|R| > .5, *p* < .01). GO enrichment analysis of these genes revealed functionally relevant biological processes, including complement activation, inflammation, and pyrimidine metabolism (Figure 5B). Complement activation and pyrimidine metabolism were associated with C1, while inflammation was related to C2.

**FIGURE 5:**
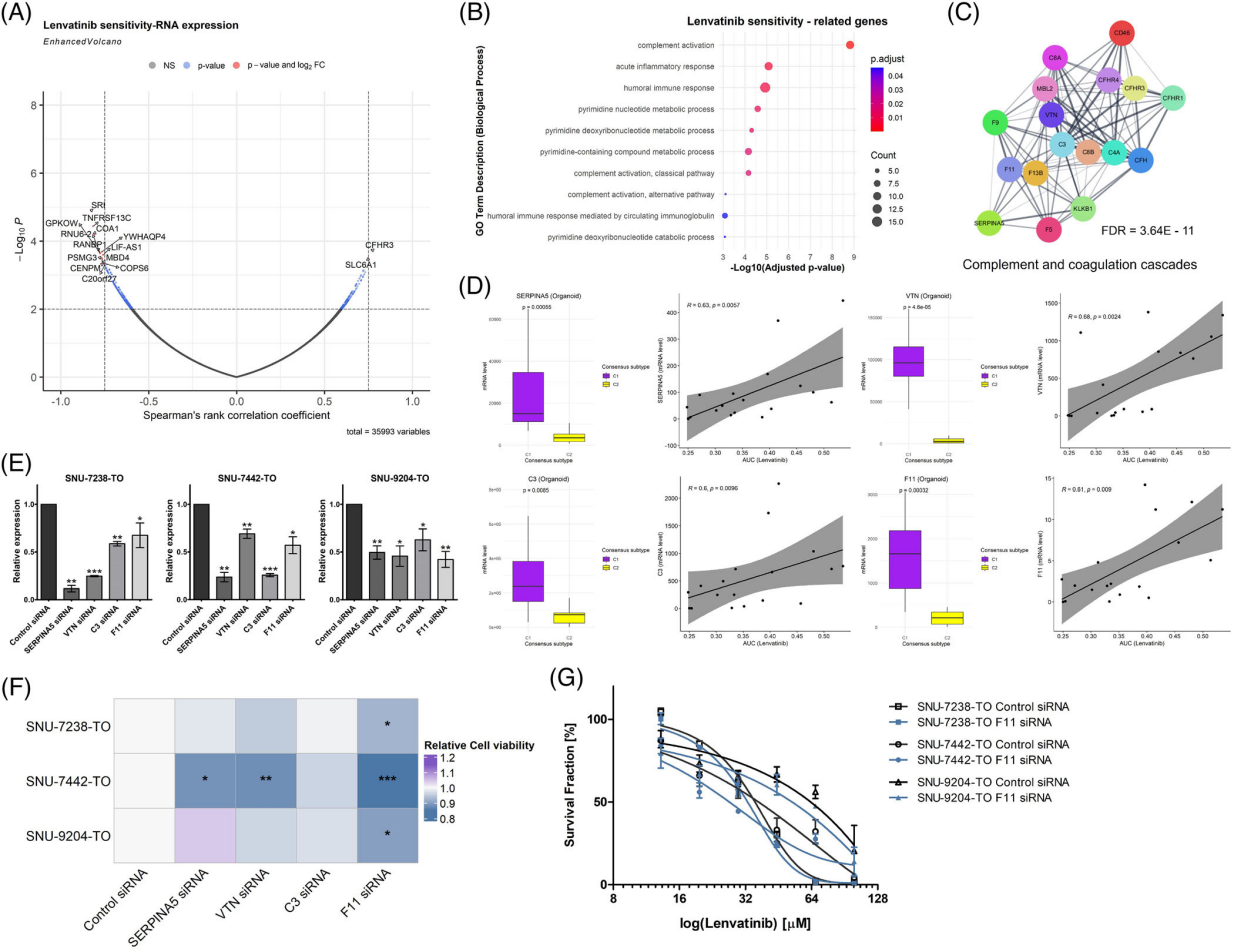
Integrative analysis of drug screening and transcriptomic data for lenvatinib sensitivity. (A) Volcano plot showing Spearman's rank correlations and their statistical significance between lenvatinib sensitivities (AUC) and mRNA expression across HCC organoids (*n* = 18). The *x*‐axis represents Spearman's rank correlation coefficient (R), and the *y*‐axis represents −log10(*p*‐value). Gray dots indicate non‐significant genes, while red and blue dots indicate genes with |*R*| > .5 and *p* < .01 and with |*R*| > .75 and *p* < .01, respectively. (B) GO enrichment analysis of 415 genes showing significant correlations with lenvatinib sensitivity (|*R*| > .5 and *p* < .01). Dot size indicates the number of genes (Count), and dot colour represents the adjusted P‐value. (C) STRING analysis showing protein–protein interactions of 17 genes within the complement and coagulation cascades pathway. (D) Left, boxplots showing mRNA expression levels of SERPINA5, VTN, C3, and F11 between C1 and C2 in SNU‐HCC organoids (*n* = 18). *p*‐values were calculated by the Wilcoxon rank‐sum test. Right, scatter plots displaying Spearman's correlation between lenvatinib sensitivities (AUC) and mRNA expressions across HCC organoids (*n* = 19, including SNU‐7941‐TO). The solid line represents the fitted regression line, and the shaded area represents the 95% CI. (E) Relative mRNA expression levels of SERPINA5, VTN, C3, and F11 in SNU‐HCC organoids following treatment with the corresponding siRNAs, as measured by real‐time qPCR. Control siRNA‐treated cells were used as the reference for relative expression. Relative gene expression levels were calculated using the 2^−ΔΔC^ *^t^* method normalized to GAPDH. Data are shown for three HCC organoids (*n* = 3), and all reactions were performed in triplicate. Statistical significance was determined using Welch's *t*‐test. (F) Heatmap showing the effects of siRNA‐mediated knockdown of SERPINA5, VTN, C3, and F11 on lenvatinib response in HCC organoids. All experiments were performed in triplicate, and relative cell viability was calculated using the lenvatinib response of control siRNA‐treated samples as the reference. The colour scale represents relative cell viability, with the ranges indicated by the corresponding colour bars. Asterisks indicate statistically significant differences compared with the control siRNA condition. *p*‐values were calculated by Welch's *t*‐test. (G) Drug‐response curves showing lenvatinib sensitivity in HCC organoids treated with control or F11 siRNA. Each curve represents an individual HCC organoid, and the response curves were compared between the control and F11 siRNA‐treated conditions. Data are presented as mean ± SD. *p < .05, **p < .01, and ***p < .001.

STRING analysis of the 415 genes further revealed that 17 genes within the complement and coagulation cascades pathway significantly interacted with each other (false discovery rate (FDR) = 3.64E – 11) (Figure 5C). Among 17 genes, SERPINA5, VTN, C3, and F11 were significantly upregulated in C1 compared with C2 (*p* < .001, Cliff's delta = .88, 95% CI: .67–.96, SERPINA5; *p* < .001, Cliff's delta = 1.00, 95% CI: 1.00–1.00, VTN; *p* = .009, Cliff's delta = .68, 95% CI: .26–.88, C3; *p* < .001, Cliff's delta = .96, 95% CI: .89–.99, F11) (Figure 5D). Notably, the expression of these genes was positively correlated with lenvatinib AUC values, indicating that higher expression was associated with increased lenvatinib resistance.

To functionally evaluate the potential roles of these genes in lenvatinib response, we performed siRNA‐mediated knockdown of SERPINA5, VTN, C3, and F11 in our HCC organoid samples, SNU‐7238‐TO, SNU‐7442‐TO, and SNU‐9204‐TO (Figure 5E). The effects of gene knockdown on cell viability following lenvatinib treatment varied among the organoid samples and among the target genes (Figure 5F). Notably, F11 knockdown consistently enhanced the response to lenvatinib across all three organoid samples. Consistent with these findings, drug‐response analysis demonstrated a reduction in cell viability following F11 knockdown compared with control siRNA treatment in all three organoid models, supporting a role for F11 in lenvatinib response (Figure 5G).

Previous reports have demonstrated that SERPINA5 reduces HCC metastasis and malignant progression,^26^ while higher expressions of C3 and F11 are associated with better survival outcomes in HCC.^27^, ^28^, ^29^ Consistently, we observed significant downregulation of these genes in C2, which exhibited poor prognosis and metastatic features.

Additionally, STRING analysis identified seven interconnected genes involved in the pyrimidine metabolism pathway (FDR = .005) (Figure S7A). Among them, DTYMK showed higher expression in C2, although the difference was not statistically significant (Figure S7B). DTYMK has been reported to be overexpressed in HCC, associated with poor prognosis and linked to drug sensitivity in HCC cell lines.^30^, ^31^, ^32^ Consistently, elevated DTYMK expression in our HCC organoids correlated with lower lenvatinib AUC values, suggesting that DTYMK may serve as a potential predictive marker for lenvatinib sensitivity.

Taken together, our integrated transcriptomic and functional analysis identified SERPINA5, VTN, C3, and F11 as candidate markers associated with lenvatinib response, with functional validation particularly supporting F11 as a potential contributor to lenvatinib resistance in HCC organoids.

### 3.7 Comparison of HBV PCR results and clinical information

HBV infection is a major contributor to HCC. Clinically, HBV infection is typically assessed by measuring serum levels of HBsAg, anti‐HBs, HBeAg, anti‐HBe, and HBV DNA. However, these markers do not indicate the presence of HBV DNA integration or cccDNA. To further evaluate tumour‐associated HBV DNA, we performed PCR targeting the HBV surface (HBVSPCR), core (HBVCPCR), and X (HBVXPCR) genes in HCC organoids, cell lines, and tissues (Figure 6).

**FIGURE 6:**
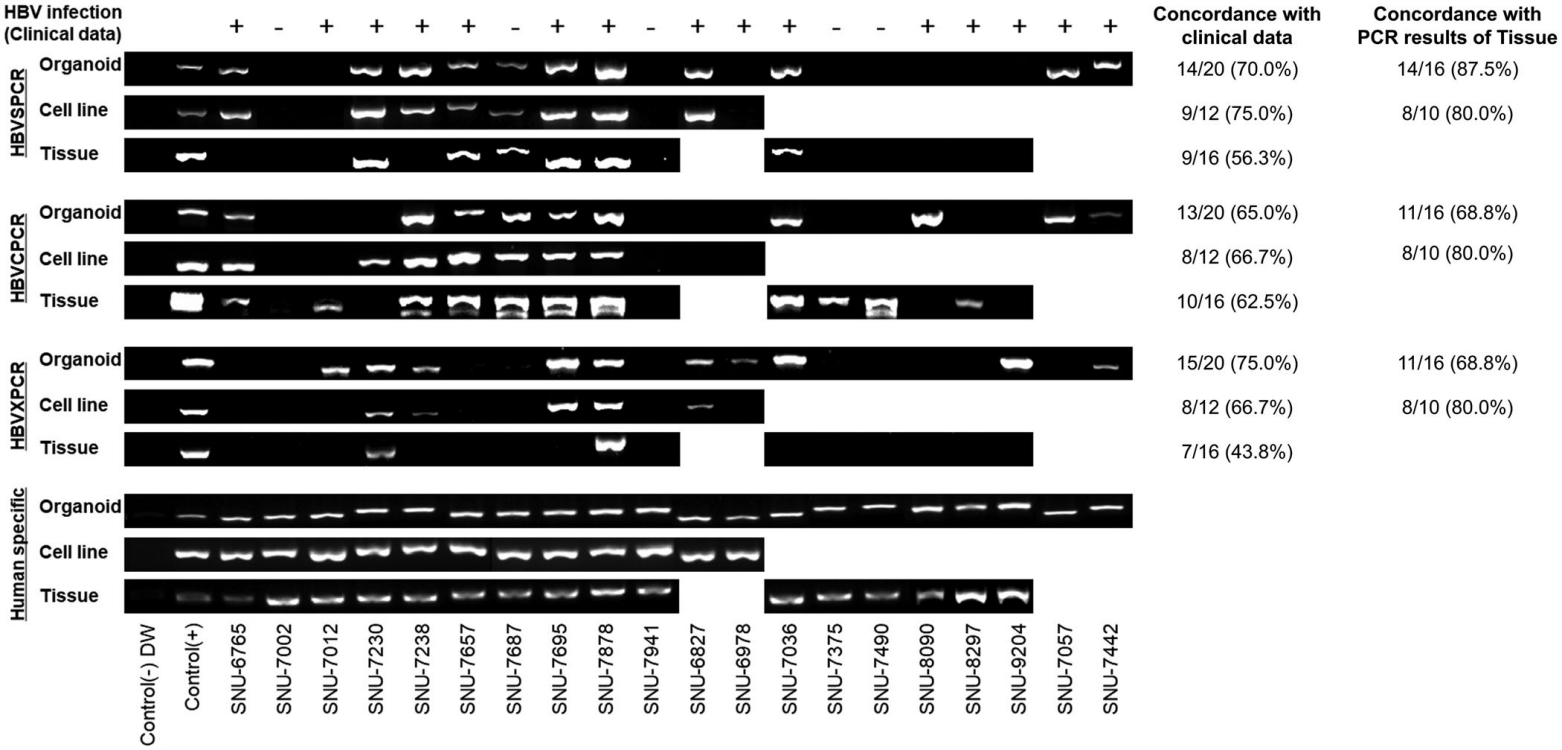
HBV RCR results of HCC‐derived organoids (*n* = 20), cell lines (*n* = 12), and paired tissues (*n* = 16). Genomic DNA extracted from each sample was subjected to PCR amplification using primers targeting the HBV core (HBVCPCR), surface (HBVSPCR), and X (HBVXPCR) genes (see also Table S2). Human‐specific primers targeting the COX1 gene were used as an internal control. Diluted water was used as a negative control, and the HBV‐positive SNU‐3058 sample was used as a positive control. The presence (+) or absence (−) of HBV infection according to the clinical data is indicated above the corresponding samples. The concordance between the PCR results of each sample and the corresponding clinical HBV infection status, as well as the concordance between organoid or cell line PCR results and those of the corresponding paired tissues, is shown on the right side.

In our cohort, the clinical HBV infection rate was 75.0% [15/20], consistent with the reported incidence of HBV‐related HCCs in Korea.^33^ However, the HBV PCR results revealed notable discrepancies with the clinical data. The concordance rates between HBV SPCR and clinical data were 70.0% [14/20], 75.0% [9/12], and 56.3% [9/16] for organoids, cell lines, and tissues, respectively. The concordance rate between organoids and paired tissues was 87.5% [14/16], while that between cell lines and paired tissue was 80.0% [8/10]. Similar discrepancies were observed for HBVCPCR and HBVXPCR. The concordance rates between HBVCPCR and clinical data were 65.0% [13/20], 66.7% [8/12], and 62.5% [10/16] for organoids, cell lines, and tissues, respectively, whereas those for HBVXPCR were 75.0% [15/20], 66.7% [8/12], and 43.8% [7/16]. The concordance rates between organoids and paired tissues were 68.8% for both HBVCPCR and HBVXPCR, and those between cell lines and paired tissues were 80.0% [8/10] for both assays.

Furthermore, we investigated the relationship between HBV status and the C1/C2 subtypes (Figure S8). Based on clinical data, HBV infection was more prevalent in C2 (90.0% [9/10]) than in C1 (62.5% [5/8]), aligning with the subtype characteristics reported in previous studies.^10^ However, no significant differences in HBV status between the subtypes were observed based on HBV PCR results in our HCC organoid cohort.

Overall, we found substantial discordance between clinical data and HBV PCR results. Also, there was no clear association between clinical HBV markers (including serum HBsAg, HBeAg, anti‐HBe, and serum HBV DNA) and PCR‐based HBV detection (Table S8). These findings should be interpreted with caution because these measurements reflect different biological layers. Clinical HBV markers mainly represent systemic viral activity or host serologic status. In contrast, PCR performed on genomic DNA from tumour tissues, organoids, or cell lines detects HBV DNA sequences retained within the tumour‐derived compartment. Therefore, the observed discordance does not indicate that PCR is superior to conventional clinical assessment, but rather suggests that PCR may provide complementary information on intra‐tumoral HBV DNA status.

### 3.8 HBV‐associated calcium signalling dysregulation is linked to oxaliplatin resistance

To investigate the effect of HBV infection at the transcriptional level, we performed differentially expressed gene (DEG) analysis based on HBV status in our HCC organoids, cell lines, and tissues (Table S9). We identified 206, 221, 252, and 190 DEGs from HBV clinical data, HBVSPCR, HBVCPCR, and HBVXPCR groups, respectively (fold change > 2.0, *p* < .05). GO enrichment analysis revealed that HBVSPCR‐related DEGs were enriched in cell adhesion‐associated biological processes, which were functionally linked to calcium signalling (Figure 7A). Similarly, HBVCPCR‐related DEGs were enriched in calcium signalling‐associated processes, including cell adhesion, development, mRNA splicing, and proliferation (Figure 7B).^34^, ^35^, ^36^, ^37^ HBVXPCR‐related DEGs were enriched in HBV‐related pathways, including detoxification/xenobiotic metabolism, lipid metabolism, oxidative stress, and inflammation (Figure 7C).^38^, ^39^, ^40^ These pathways were also associated with calcium signalling.^41^, ^42^ In addition, post‐synaptic density assembly, another process linked to calcium signalling,^43^ was enriched in the HBVXPCR group. STRING‐based protein–protein interaction analysis demonstrated that HBV status‐related DEGs converged on interconnected networks associated with calcium pathways (Figure 7D). Consistent with previous reports that HBV infection induces extracellular Ca^2^^+^ influx,^44^, ^45^ these results indicate a differential enrichment of calcium signalling‐related pathways depending on HBV status.

**FIGURE 7:**
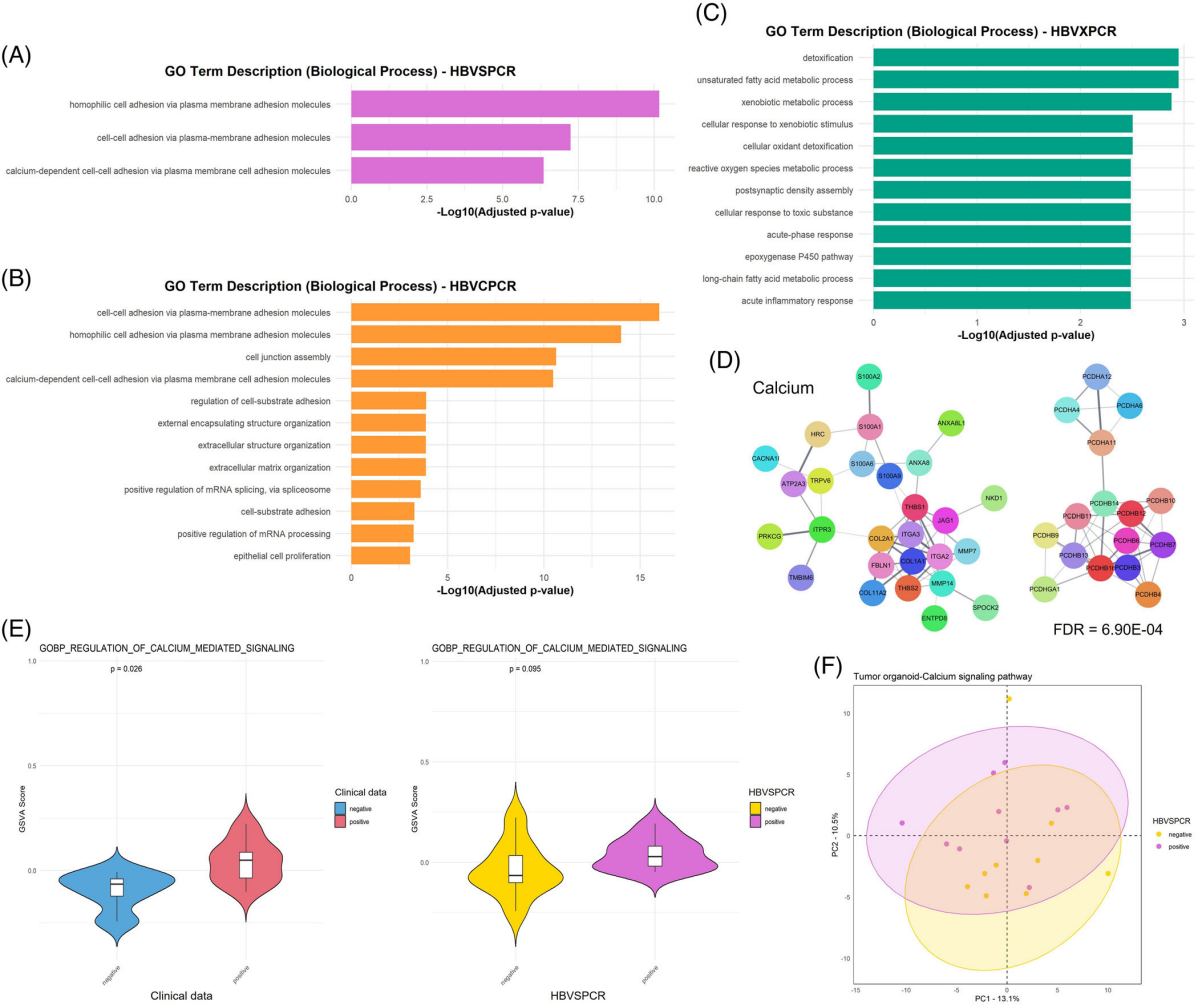
Association between HBV infection and calcium signalling pathways. (A–C) GO enrichment analysis of (A) HBVSPCR‐related 221 DEGs (*p* < .05), (B) HBVCPCR‐related 252 DEGs (*p* < .05), and (C) HBVXPCR‐related 190 DEGs (*p* < .05). The *x*‐axis represents −log10(adjusted *p*‐value). (D) STRING analysis showing protein–protein interaction networks of genes associated with calcium‐related pathways among HBV status‐related DEGs. (E) Violin plots representing GSVA enrichment scores of the GOBP_REGULATION_OF_CALCIUM_MEDIATED_SIGNALING pathway in HCC‐derived organoids (*n* = 19) by HBV infection status in (left) clinical data and (right) HBVSPCR. Colours indicate HBV‐negative and HBV‐positive samples. *p*‐values were calculated by the Wilcoxon rank‐sum test. (F) PCA plot showing the clustering of HCC organoids (*n* = 19) by HBVSPCR status based on the expression of calcium signalling pathway‐related genes from the KEGG database. Colours indicate HBVSPCR‐negative and HBVSPCR‐positive samples. The x‐axis indicates PC1 (13.1% variance), and the *y*‐axis indicates PC2 (10.5% variance).

To validate this observation, GSVA scores for calcium‐mediated signalling pathway were calculated in our HCC organoids: Regulation of calcium‐mediated signalling was significantly higher in the HBV‐positive group compared with the HBV‐negative group within both the clinical data and HBVSPCR cohorts (*p* = .026, Cliff's delta = −.69, 95% CI: −.89 to −.23, clinical data; *p* = .095, Cliff's delta = −.47, 95% CI: −.78 to .03, HBVSPCR) (Figure 7E; Figure S9A). Furthermore, PCA showed a clear separation between HBVSPCR‐positive and HBVSPCR‐negative samples (Figure 7F; Figure S9B).

Next, we assessed the effect of HBV status on drug response (Figure 8A; Figure S9C,D). Among the agents tested, oxaliplatin exhibited a significantly increased resistance in HBV‐positive group, as evidenced by both clinical data and HBVSPCR (*p* = .046, Cliff's delta = −.63, 95% CI: −.88 to −.13, clinical data; *p* = .031, Cliff's delta = −.60, 95% CI: −.85 to −.15, HBVSPCR) (Figure 8A). Moreover, oxaliplatin AUC values positively correlated with the GSVA scores for the calcium‐mediated signalling pathway (Figure 8B), indicating a link between calcium signalling activation and oxaliplatin resistance in HBV‐positive HCC.

**FIGURE 8:**
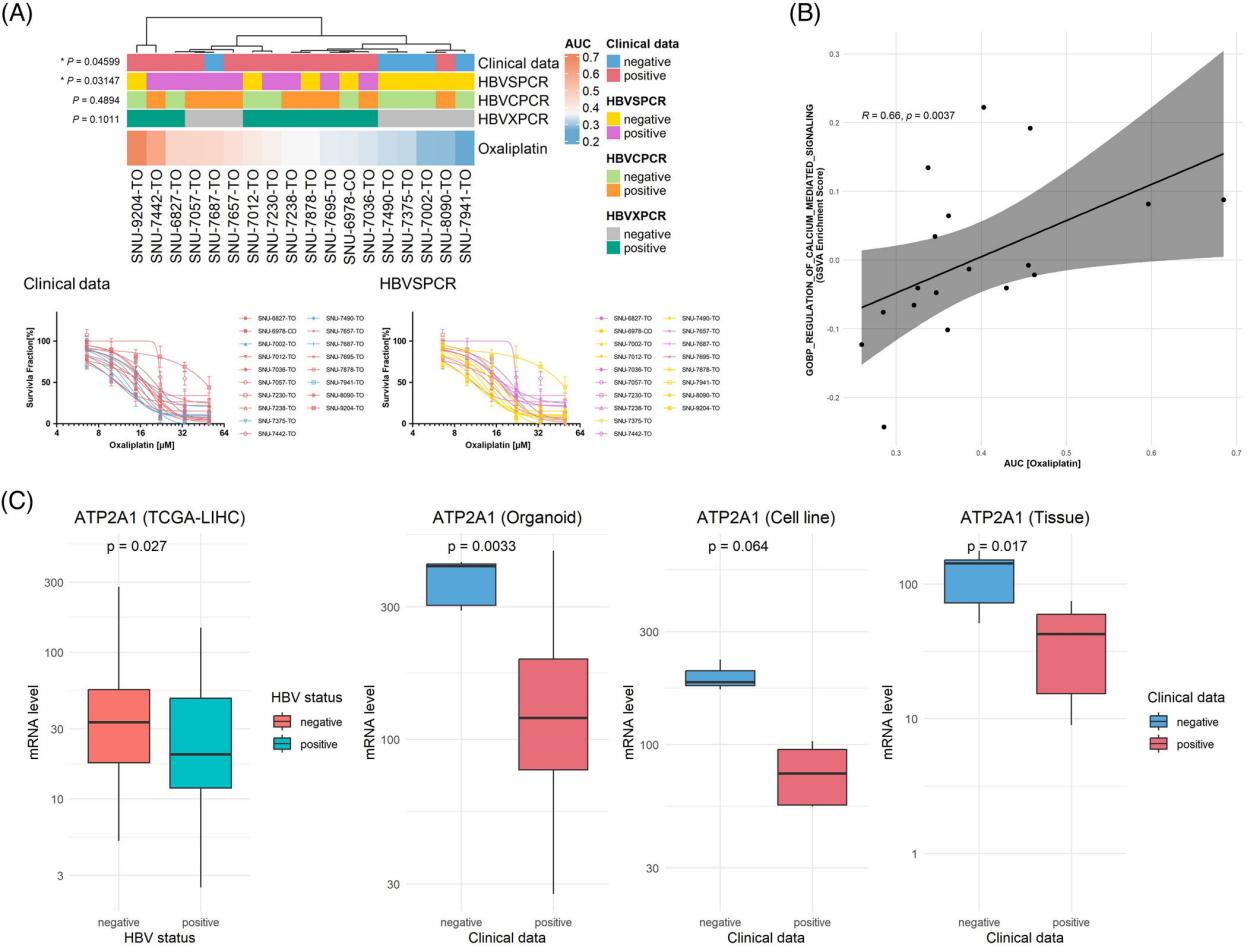
Association of oxaliplatin response with HBV infection through calcium signalling pathways. (A) Top, heatmap showing oxaliplatin response across HCC organoids (*n* = 18), with the colour annotation representing the HBV status based on clinical data, HBVSPCR, HBVCPCR, and HBVXPCR. The colour scales represent AUC values, with the ranges indicated by the corresponding colour bars. *p*‐values indicate comparisons of oxaliplatin AUC values between HBV‐negative and HBV‐positive groups and were calculated by the Wilcoxon rank‐sum test. Bottom, the results of drug responses by HBV clinical data and HBVSPCR are displayed in the form of drug‐response curves (*n* = 18). Data are presented as mean ± SD. (B) Scatter plots displaying Spearman's correlation between oxaliplatin sensitivities (AUC) and GSVA enrichment scores of the GOBP_REGULATION_OF_CALCIUM_MEDIATED_SIGNALING pathway in HCC organoids (*n* = 18). (C) Boxplots showing mRNA expression levels of ATP2A1 in the TCGA‐LIHC cohort (*n* = 191), HCC‐derived organoids (*n* = 19), cell lines (*n* = 12), and paired tissue samples (*n* = 16) by HBV status. *p*‐values were calculated by the Wilcoxon rank‐sum test. *p < .05.

We then examined calcium signalling‐related genes derived from the Kyoto Encyclopedia of Genes and Genomes (KEGG) database. Although several genes were significantly differentially expressed by HBV status in the TCGA‐LIHC cohort (Table S10),^46^ only ATP2A1 was consistently downregulated in clinical HBV‐positive groups compared with clinical HBV‐negative groups across our organoid, cell line, and tissue samples (*p* = .027, Cliff's delta = .22, 95% CI: .03−.40, TCGA‐LIHC; *p* = .003, Cliff's delta = .86, 95% CI: .59–.96, organoids; *p* = .064, Cliff's delta = .78, 95% CI: .25–.95, cell lines; *p* = .017, Cliff's delta = .78, 95% CI: .40–.93, tissues) (Figure 8C). ATP2A1 encodes a sarcoplasmic/endoplasmic reticulum Ca^2^^+^‐ATPase (SERCA), which pumps Ca^2^^+^ from the cytoplasm into the sarcoplasmic reticulum lumen, thereby lowering cytoplasmic Ca^2^^+^ levels. Notably, SERCA inhibition was previously shown to enhance HBV transcription.^47^ Together with previous findings that HBV infection increases cytoplasmic Ca^2^^+^ concentration,^44^, ^45^, ^48^ ATP2A1 downregulation may be associated with HBV‐related calcium dysregulation, although this relationship remains to be experimentally tested.

Collectively, these findings support a hypothetical model in which HBV‐related calcium dysregulation may contribute to oxaliplatin resistance. HBV infection has been reported to elevate cytoplasmic Ca^2^^+^ concentration,^44^, ^45^, ^48^ while oxaliplatin relies on oxalate‐mediated extracellular Ca^2^^+^ chelation for effective metabolism. Han et al.^49^ reported that calcium increased oxaliplatin metabolism, and Morimoto et al.^50^ reported that elevated intracellular Ca^2^^+^ concentration resulted in platinum resistance.^50^ These findings raise the possibility that the HBV‐induced reduction of extracellular Ca^2^^+^ may hinder oxaliplatin metabolism and thereby diminish its therapeutic efficacy. In line with this hypothesis, ATP2A1 was consistently downregulated in HBV‐positive samples, suggesting that HBV infection may dysregulate calcium signalling in part through reduced ATP2A1 expression. Thus, our findings provide a potential mechanistic framework linking HBV status, calcium signalling, and oxaliplatin resistance, which warrants further functional investigation.

## 4 DISCUSSION

In this study, we established and comprehensively characterized patient‐derived HCC models using genomic, transcriptomic, pharmacological, and HBV PCR analyses. These models faithfully recapitulated the molecular and pathological features of HCC, providing a robust platform for exploring HCC biology and potential biomarkers.

Our integrative analysis revealed two distinct molecular subtypes of HCC organoids. C1 was enriched in metabolic and coagulation‐related pathways with a more favourable prognosis, while C2 showed enrichment in inflammation‐, autophagy‐, and metastasis‐related pathways with a poorer prognosis. This classification largely recapitulates previously reported metabolic and proliferative HCC subtyping frameworks.^10^, ^11^, ^12^, ^13^, ^15^, ^16^, ^17^, ^18^ Moreover, we derived 92 signature genes from the organoid models and validated their ability to reproduce the C1/C2 classification in the independent TCGA‐LIHC cohort, supporting the reproducibility of these molecular features. Our study extends these established molecular features to patient‐derived HCC organoids and provides additional information on their pharmacological and biological characteristics. In particular, the C1/C2 subtypes exhibited distinct drug‐response profiles, with C2 showing greater sensitivity to everolimus and lenvatinib and higher enrichment of the PI3K/AKT/mTOR pathway. These findings add a pharmacological dimension to established HCC molecular subtypes and suggest that subtype‐associated biology may contribute to differential therapeutic responses.

In addition, our integrated transcriptomic and drug‐response analyses identified candidate genes associated with lenvatinib response. Complement and coagulation‐related genes, including SERPINA5, VTN, C3, and F11, were correlated with resistance to lenvatinib, whereas the pyrimidine metabolism‐related gene DTYMK was associated with increased sensitivity. Although these genes are not canonical drivers of HCC, prior studies have implicated them in tumour progression, immune regulation, and drug response.^26^, ^27^, ^28^, ^30^, ^31^, ^32^ Additionally, SERPINA5, VTN, and F11 were among the 92 signature genes, suggesting their relevance to the molecular characteristics of the C1/C2 subtypes and lenvatinib response. Notably, siRNA‐mediated knockdown of F11 consistently enhanced the response to lenvatinib across three HCC organoid models, providing functional support for its potential role in lenvatinib response. These findings suggest that these genes, particularly F11, warrant further investigation as potential determinants of lenvatinib response in HCC.

HBV infection remains one of the key drivers of HCC. We observed substantial discrepancies between clinical data and PCR‐based HBV detection, which may reflect both biological and technical factors. Clinically HBV‐negative but PCR‐positive cases may represent occult HBV infection, residual or integrated HBV DNA, or retained episomal viral DNA such as cccDNA. Conversely, clinically HBV‐positive but PCR‐negative cases may result from spatial tumour heterogeneity, viral integration events lacking the PCR‐targeted surface, core, or X regions, or loss of HBV‐containing subclones during the establishment of organoids or cell lines. Importantly, our PCR assay cannot distinguish integrated HBV DNA from cccDNA or other retained viral DNA forms. Therefore, PCR positivity in this study should be interpreted as evidence of retained HBV DNA sequences in tumour‐derived samples, rather than as direct proof of HBV integration. Future studies using HBV capture sequencing or cccDNA‐specific assays will be required to define the molecular forms and functional roles of HBV DNA in HCC‐derived models.

Furthermore, transcriptomic analysis revealed an enrichment of calcium signalling pathways in HBV‐positive samples, consistent with previous reports that HBV infection perturbs Ca^2^^+^ homeostasis.^44^, ^45^, ^48^ Moreover, HBV‐positive samples exhibited greater resistance to oxaliplatin, whose cytotoxicity depends on oxalate‐mediated Ca^2^^+^ chelation. We identified ATP2A1, which encodes a SERCA, as a potential biomarker associated with HBV status and calcium signalling. Together, these findings suggest a potential association between HBV infection, calcium signalling, and oxaliplatin response, providing a hypothesis‐generating framework for further investigation of the relationship between viral infection and chemotherapy response.

This study has several limitations. First, the number of patient‐derived models was relatively small, which limited the statistical power for detecting subtype‐specific differences or drug responses. Accordingly, some of the observed associations related to mutation status or HBV status, as well as certain subgroup comparisons, should be interpreted with caution and require validation in larger, independent cohorts. Second, although the organoids showed transcriptomic characteristics representative of their corresponding parental tumours, longitudinal changes in molecular and morphological features during serial passaging were not systematically assessed. Therefore, the possibility of clonal selection or phenotypic drift during extended culture cannot be completely excluded. Third, the mechanistic relationships proposed in this study, particularly those linking viral infection, calcium dysregulation, and oxaliplatin sensitivity, were not experimentally validated and warrant further functional investigation.

Despite these limitations, this study provides one of the most comprehensive integrative characterizations of patient‐derived HCC models. By combining genomic, transcriptomic, pharmacological, and HBV‐associated analyses, we characterized molecular subtypes, identified candidate determinants of drug response, and revealed potential associations between HBV infection and chemotherapy resistance. Collectively, these findings advance our understanding of HCC biology and highlight the value of organoid models as preclinical platforms. The established organoids and cell lines in this study will be deposited in the Korean Organoid Biobank (https://organoid.snu.ac.kr) and the Korean Cell Line Bank (https://cellbank.snu.ac.kr), and sequencing data will also be registered with the K‐BDS (https://kbds.re.kr/). These resources will support domestic and international researchers and contribute to the development of precision medicine strategies for HCC.

## 5 CONCLUSION

In this study, we present newly established HCC patient‐derived organoid models with comprehensive molecular characterization and drug sensitivity profiling. These models faithfully recapitulate the molecular features of HCC and enable the characterization of molecular subtypes through integrated genomic, transcriptomic, pharmacological, and HBV‐associated analyses. Our findings provide additional insights into HCC molecular heterogeneity, drug response, and HBV‐associated biology.

## AUTHOR CONTRIBUTIONS

S.‐H.K.: conceptualization; methodology, resources, formal analysis, data curation, software, visualization, validation, investigation, funding acquisition, writing—original draft, project administration, writing—review and editing. Y.R.C.: conceptualization, data curation, formal analysis, writing—original draft, and writing—review and editing. E.W.C.: resources, methodology, and investigation. S.H.K.: resources, methodology, and investigation. S.C.O.: resources, methodology, and investigation. J.Y.K.: data curation, investigation, and writing—review and editing. S.H.: data curation, investigation, and writing—review and editing. S.K.H.: data curation, investigation, and writing—review and editing. K.‐W.L.: conceptualization, writing—review and editing, and supervision. J.‐L.K.: project administration, writing—review and editing, supervision, funding acquisition.

## ETHICS APPROVAL AND CONSENT TO PARTICIPATE

This study was approved by the Institutional Review Board of Seoul National University Hospital (IRB no. 1102‐098‐357). The study was performed in accordance with the Declaration of Helsinki. Written informed consent was obtained from all patients enrolled in this study.

## CONSENT FOR PUBLICATION

Informed consent was obtained from all subjects involved in the study.

## CONFLICT OF INTEREST STATEMENT

The authors declare no conflict of interest.

## Supporting information



Supporting information

Supporting information

## Data Availability

The mRNA sequencing data and corresponding clinical information of the TCGA‐LIHC database were retrieved from https://tcga‐data.nci.nih.gov/tcga/. The dataset generated from the use or analysis in this study can be obtained from the corresponding author upon reasonable request.

## References

[1] Sung H , Ferlay J , Siegel RL , et al. Global Cancer Statistics 2020: GLOBOCAN estimates of incidence and mortality worldwide for 36 cancers in 185 countries. CA Cancer J Clin. 2021;71(3):209‐249. doi:10.3322/caac.21660 10.3322/caac.21660 33538338

[2] McGlynn KA , Petrick JL , El‐Serag HB . Epidemiology of Hepatocellular Carcinoma. Hepatology. 2021;73 (Suppl 1):4‐13. doi:10.1002/hep.31288 10.1002/hep.31288 PMC7577946 32319693

[3] Toh MR , Wong EYT , Wong SH , et al. Global epidemiology and genetics of hepatocellular carcinoma. Gastroenterology. 2023;164(5):766‐782. doi:10.1053/j.gastro.2023.01.033 10.1053/j.gastro.2023.01.033 36738977

[4] Yang J , Pan G , Guan L , et al. The burden of primary liver cancer caused by specific etiologies from 1990 to 2019 at the global, regional, and national levels. Cancer Med. 2022;11(5):1357‐1370. doi:10.1002/cam4.4530 10.1002/cam4.4530 PMC8894689 34989144

[5] Zhou Y , Peng Y , Yuan H , Long Z , Wu S , Yang J . Serum hepatitis B virus ribonucleic acid and its influencing factors in chronic hepatitis B. Adv Clin Exp Med. 2021;30(4):361‐367. doi:10.17219/acem/131756 10.17219/acem/131756 33928768

[6] Taddese M , Grudda T , Belluccini G , et al. Transcription of hepatitis B surface antigen shifts from cccDNA to integrated HBV DNA during treatment. J Clin Invest. 2025;135(6):e184243. doi:10.1172/JCI184243 10.1172/JCI184243 PMC11910228 39898797

[7] Li M , Wu S , Luo H , et al. HBV DNA integration gene CCDC91 is oncogenic and a potential therapeutic target for hepatocellular carcinoma. Commun Biol. 2025;8(1):1079. doi:10.1038/s42003‐025‐08369‐1 10.1038/s42003-025-08369-1 PMC12276357 40683971

[8] Xian L , Zhao P , Chen X , et al. Heterogeneity, inherent and acquired drug resistance in patient‐derived organoid models of primary liver cancer. Cell Oncol (Dordr). 2022;45(5):1019‐1036. doi:10.1007/s13402‐022‐00707‐3 10.1007/s13402-022-00707-3 PMC12978112 36036881

[9] Nuciforo S , Fofana I , Matter MS , et al. Organoid models of human liver cancers derived from tumor needle biopsies. Cell Rep. 2018;24(5):1363‐1376. doi:10.1016/j.celrep.2018.07.001 10.1016/j.celrep.2018.07.001 PMC6088153 30067989

[10] Caruso S , Calatayud AL , Pilet J , et al. Analysis of liver cancer cell lines identifies agents with likely efficacy against hepatocellular carcinoma and markers of response. Gastroenterology. 2019;157(3):760‐776. doi:10.1053/j.gastro.2019.05.001 10.1053/j.gastro.2019.05.001 31063779

[11] Lee JS , Chu IS , Heo J , et al. Classification and prediction of survival in hepatocellular carcinoma by gene expression profiling. Hepatology. 2004;40(3):667‐676. doi:10.1002/hep.20375 10.1002/hep.20375 15349906

[12] Hoshida Y , Nijman SM , Kobayashi M , et al. Integrative transcriptome analysis reveals common molecular subclasses of human hepatocellular carcinoma. Cancer Res. 2009;69(18):7385‐7392. doi:10.1158/0008‐5472.CAN‐09‐1089 10.1158/0008-5472.CAN-09-1089 PMC3549578 19723656

[13] Ji S , Feng L , Fu Z , et al. Pharmaco‐proteogenomic characterization of liver cancer organoids for precision oncology. Sci Transl Med. 2023;15(706):eadg3358. doi:10.1126/scitranslmed.adg3358 10.1126/scitranslmed.adg3358 PMC10949980 37494474

[14] Petitprez F , Meunier L , Letouzé E , et al. liverK: an R package for transcriptome‐based computation of molecular subtypes and functional signatures in liver cancer. bioRxiv. 2019:540005. doi:10.1101/540005

[15] Lee SH , Yim SY , Jeong YS , et al. Consensus subtypes of hepatocellular carcinoma associated with clinical outcomes and genomic phenotypes. Hepatology. 2022;76(6):1634‐1648. doi:10.1002/hep.32490 10.1002/hep.32490 PMC9519807 35349735

[16] Gao Q , Zhu H , Dong L , et al. Integrated proteogenomic characterization of HBV‐related hepatocellular carcinoma. Cell. 2019;179(5):1240. doi:10.1016/j.cell.2019.10.038 10.1016/j.cell.2019.10.038 31730861

[17] Xing X , Hu E , Ouyang J , et al. Integrated omics landscape of hepatocellular carcinoma suggests proteomic subtypes for precision therapy. Cell Rep Med. 2023;4(12):101315. doi:10.1016/j.xcrm.2023.101315 10.1016/j.xcrm.2023.101315 PMC10783603 38091986

[18] Jiang Y , Sun A , Zhao Y , et al. Proteomics identifies new therapeutic targets of early‐stage hepatocellular carcinoma. Nature. 2019;567(7747):257‐261. doi:10.1038/s41586‐019‐0987‐8 10.1038/s41586-019-0987-8 30814741

[19] Chen H , Liu H , Qing G . Targeting oncogenic Myc as a strategy for cancer treatment. Signal Transduct Target Ther. 2018;3:5. doi:10.1038/s41392‐018‐0008‐7 10.1038/s41392-018-0008-7 PMC5837124 29527331

[20] Peixoto E , Richard S , Pant K , Biswas A , Gradilone SA . The primary cilium: its role as a tumor suppressor organelle. Biochem Pharmacol. 2020;175:113906. doi:10.1016/j.bcp.2020.113906 10.1016/j.bcp.2020.113906 PMC7231466 32169416

[21] Lee KH . Primary cilia: a novel research approach to overcome anticancer drug resistance. Front Mol Biosci. 2023;10:1270639. doi:10.3389/fmolb.2023.1270639 10.3389/fmolb.2023.1270639 PMC10602908 37900915

[22] Della Corte CM , Viscardi G , Papaccio F , et al. Implication of the Hedgehog pathway in hepatocellular carcinoma. World J Gastroenterol. 2017;23(24):4330‐4340. doi:10.3748/wjg.v23.i24.4330 10.3748/wjg.v23.i24.4330 PMC5487497 28706416

[23] Cheng WT , Xu K , Tian DY , Zhang ZG , Liu LJ , Chen Y . Role of Hedgehog signaling pathway in proliferation and invasiveness of hepatocellular carcinoma cells. Int J Oncol. 2009;34(3):829‐836. doi:10.3892/ijo_00000209 10.3892/ijo_00000209 19212688

[24] Chen Y , Dai S , Cheng CS , Chen L . Lenvatinib and immune‐checkpoint inhibitors in hepatocellular carcinoma: mechanistic insights, clinical efficacy, and future perspectives. J Hematol Oncol. 2024;17(1):130. doi:10.1186/s13045‐024‐01647‐1 10.1186/s13045-024-01647-1 PMC11663365 39709431

[25] Huang MY , Hu SY , Dong J , et al. The DRAP1/DR1 repressor complex increases mTOR activity to promote progression and confer everolimus sensitivity in triple‐negative breast cancer. Cancer Res. 2024;84(16):2660‐2673. doi:10.1158/0008‐5472.CAN‐23‐2781 10.1158/0008-5472.CAN-23-2781 38748783

[26] Jing Y , Jia D , Wong CM , et al. SERPINA5 inhibits tumor cell migration by modulating the fibronectin‐integrin beta1 signaling pathway in hepatocellular carcinoma. Mol Oncol. 2014;8(2):366‐377. doi:10.1016/j.molonc.2013.12.003 10.1016/j.molonc.2013.12.003 PMC5528558 24388360

[27] Zhao SS , Bai RR , Zhang BH , et al. Investigating the diagnostic and prognostic significance of genes related to fatty acid metabolism in hepatocellular carcinoma. BMC Gastroenterol. 2024;24(1):409. doi:10.1186/s12876‐024‐03495‐2 10.1186/s12876-024-03495-2 PMC11566841 39548390

[28] Qian X , Yang Z , Gao L , Liu Y , Yan J . The role of complement in the clinical course of hepatocellular carcinoma. Immun Inflamm Dis. 2022;10(3):e569. doi:10.1002/iid3.569 10.1002/iid3.569 PMC8926509 34813686

[29] Su H , Chen Y , Wang W . Novel prognostic model of complement and coagulation cascade‐related genes correlates with immune environment and drug sensitivity in hepatocellular carcinoma. Heliyon. 2024;10(19):e38230. doi:10.1016/j.heliyon.2024.e38230 10.1016/j.heliyon.2024.e38230 PMC11466567 39391504

[30] Sun F , Liu Y , Gong T , et al. Inhibition of DTYMK significantly restrains the growth of HCC and increases sensitivity to oxaliplatin. Cell Death Dis. 2021;12(12):1093. doi:10.1038/s41419‐021‐04375‐3 10.1038/s41419-021-04375-3 PMC8602592 34795209

[31] Cai LY , Chen SJ , Xiao SH , et al. Targeting p300/CBP attenuates hepatocellular carcinoma progression through epigenetic regulation of metabolism. Cancer Res. 2021;81(4):860‐872. doi:10.1158/0008‐5472.CAN‐20‐1323 10.1158/0008-5472.CAN-20-1323 33361394

[32] Guo Y , Luo W , Huang S , et al. DTYMK expression predicts prognosis and chemotherapeutic response and correlates with immune infiltration in hepatocellular carcinoma. J Hepatocell Carcinoma. 2021;8:871‐885. doi:10.2147/JHC.S312604 10.2147/JHC.S312604 PMC8349219 34377684

[33] Shim JJ , Kim JW , Kim BH . Risk of hepatitis B virus‐related hepatocellular carcinoma development is much higher in Koreans than in Taiwanese. Korean J Intern Med. 2017;32(5):940‐942. doi:10.3904/kjim.2016.180 10.3904/kjim.2016.180 PMC5583456 28870014

[34] Xie J . Control of alternative pre‐mRNA splicing by Ca(++) signals. Biochim Biophys Acta. 2008;1779(8):438‐452. doi:10.1016/j.bbagrm.2008.01.003 10.1016/j.bbagrm.2008.01.003 PMC3500379 18258215

[35] Trebak M , Kinet JP . Calcium signalling in T cells. Nat Rev Immunol. 2019;19(3):154‐169. doi:10.1038/s41577‐018‐0110‐7 10.1038/s41577-018-0110-7 PMC6788797 30622345

[36] Brodskiy PA , Zartman JJ . Calcium as a signal integrator in developing epithelial tissues. Phys Biol. 2018;15(5):051001. doi:10.1088/1478‐3975/aabb18 10.1088/1478-3975/aabb18 PMC5993055 29611534

[37] van Roy F , Berx G . The cell‐cell adhesion molecule E‐cadherin. Cell Mol Life Sci. 2008;65(23):3756‐3788. doi:10.1007/s00018‐008‐8281‐1 10.1007/s00018-008-8281-1 PMC11131785 18726070

[38] Schollmeier A , Glitscher M , Hildt E . Relevance of HBx for hepatitis B virus‐associated pathogenesis. Int J Mol Sci. 2023;24(5):4964. doi:10.3390/ijms24054964 10.3390/ijms24054964 PMC10003785 36902395

[39] Yan Y , Wei Z , Zheng M , Lu M , Wang X . HBV and host metabolic crosstalk: reprogramming pathways for viral replication and pathogenesis. Virol Sin. 2025;40(5):685‐693. doi:10.1016/j.virs.2025.09.008 10.1016/j.virs.2025.09.008 PMC12665413 41022265

[40] Zhang A , Sun H , Han Y , Yan G , Wang X . Urinary metabolic biomarker and pathway study of hepatitis B virus infected patients based on UPLC‐MS system. PLoS One. 2013;8(5):e64381. doi:10.1371/journal.pone.0064381 10.1371/journal.pone.0064381 PMC3655955 23696887

[41] Arruda AP , Hotamisligil GS . Calcium homeostasis and organelle function in the pathogenesis of obesity and diabetes. Cell Metab. 2015;22(3):381‐397. doi:10.1016/j.cmet.2015.06.010 10.1016/j.cmet.2015.06.010 PMC4558313 26190652

[42] Humbert A , Lefebvre R , Nawrot M , Caussy C , Rieusset J . Calcium signalling in hepatic metabolism: health and diseases. Cell Calcium. 2023;114:102780. doi:10.1016/j.ceca.2023.102780 10.1016/j.ceca.2023.102780 37506596

[43] Shah KD , Zhang Y . Calcium signaling and postsynaptic density dynamics: the roles of calmodulin in synaptic protein regulation. Front Mol Neurosci. 2026;19:1831452. doi:10.3389/fnmol.2026.1831452 10.3389/fnmol.2026.1831452 PMC13269105 42311417

[44] Kong F , Zhang F , Liu X , et al. Calcium signaling in hepatitis B virus infection and its potential as a therapeutic target. Cell Commun Signal. 2021;19(1):82. doi:10.1186/s12964‐021‐00762‐7 10.1186/s12964-021-00762-7 PMC8349099 34362380

[45] Casciano JC , Duchemin NJ , Lamontagne RJ , Steel LF , Bouchard MJ . Hepatitis B virus modulates store‐operated calcium entry to enhance viral replication in primary hepatocytes. PLoS One. 2017;12(2):e0168328. doi:10.1371/journal.pone.0168328 10.1371/journal.pone.0168328 PMC5289456 28151934

[46] Cancer Genome Atlas Research Network . Comprehensive and integrative genomic characterization of hepatocellular carcinoma. Cell. 2017;169(7):1327–1341 e23. doi:10.1016/j.cell.2017.05.046 10.1016/j.cell.2017.05.046 PMC5680778 28622513

[47] Chen H , Mu M , Liu Q , et al. Hepatocyte endoplasmic reticulum stress inhibits hepatitis B virus secretion and delays intracellular hepatitis B virus clearance after entecavir treatment. Front Med (Lausanne). 2020;7:589040. doi:10.3389/fmed.2020.589040 10.3389/fmed.2020.589040 PMC7890007 33614671

[48] Bouchard MJ , Wang LH , Schneider RJ . Calcium signaling by HBx protein in hepatitis B virus DNA replication. Science. 2001;294(5550):2376‐2378. doi:10.1126/science.294.5550.2376 10.1126/science.294.5550.2376 11743208

[49] Han CH , Khwaounjoo P , Hill AG , Miskelly GM , McKeage MJ . Predicting effects on oxaliplatin clearance: in vitro, kinetic and clinical studies of calcium‐ and magnesium‐mediated oxaliplatin degradation. Sci Rep. 2017;7(1):4073. doi:10.1038/s41598‐017‐04383‐4 10.1038/s41598-017-04383-4 PMC5481441 28642473

[50] Morimoto A , Serada S , Enomoto T , et al. Annexin A4 induces platinum resistance in a chloride‐and calcium‐dependent manner. Oncotarget. 2014;5(17):7776‐7787. doi:10.18632/oncotarget.2306 10.18632/oncotarget.2306 PMC4202160 25277200

