# Supplementary material for: Integrative characterization of human hepatocellular carcinoma‐derived organoids and their HBV‐associated biology
Source: Clin Transl Med. 2026 Oct 6;16(10):e70853. doi: 10.1002/ctm2.70853 (PMC13639540; doi:10.1002/ctm2.70853)
Supplement: Supplementary file 1 [file CTM2-16-e70853-s001.zip › Supplementary table/Supplementary Table 2-3, 5-6, and 8-9_Revised.docx]

Supplementary Table 2. The detailed PCR conditions and primer sequences.

| Target gene | Forward primer (5' to 3') | Reverse primer (5' to 3') | Annealing temperature | Extension time |
| --- | --- | --- | --- | --- |
| HBV S | TCACCATATTCTTGGGAACAAGA | CGAACCACTGAACAAATGGC | 55℃ | 60 sec |
| HBV C | AAGCTGTGCCTTGGGTGGCTT | CGAGATTGAGATCTTCTGCGAC | 68℃ | 40 sec |
| HBV X | ATGGCTGCTAGGCTGTGCTGCCAAC | TTAGGCAGAGGTGAAAAAGTTGCAT | 55℃ | 30 sec |
| COX1 | TTCGGCGCATGAGCTGGAGTCC | TATGCGGGGAAACGCCATATCG | 58℃ | 30 sec |

Supplementary Table 3. List of established patient-derived organoids, cell lines, and paired tissues used in this study.

| **Group** | **SNU No.** | **Sex/Age** | **Sample type** | **Passage (Whole-exome sequencing)** | **Passage**  **(RNA sequencing)** |
| --- | --- | --- | --- | --- | --- |
| 1 | SNU-6765-TO | Male/57 | Cancer organoid | 6 | 7 |
|  | SNU-6765T | Male/57 | Cancer cell line | 10 | 11 |
|  | SNU-6765NT | Male/57 | Normal tissue | NA | NA |
|  | SNU-6765CT | Male/57 | Cancer tissue | NA | NA |
| 2 | SNU-6827-TO | Male/40 | Cancer organoid | 4 | 7 |
|  | SNU-6827T | Male/40 | Cancer cell line | 10 | 10 |
| 3 | SNU-6978 | Male/51 | Cancer cell line | 7 | 8 |
|  | SNU-6978-CO | Male/51 | Cancer cell line to organoid | 6 | 6 |
| 4 | SNU-7002-TO | Male/66 | Cancer organoid | 4 | 6 |
|  | SNU-7002T | Male/66 | Cancer cell line | 10 | 10 |
|  | SNU-7002NT | Male/66 | Normal tissue | NA | NA |
|  | SNU-7002CT | Male/66 | Cancer tissue | NA | NA |
| 5 | SNU-7012-TO | Female/38 | Cancer organoid | 3 | 7 |
|  | SNU-7012T | Female/38 | Cancer cell line | 8 | 10 |
|  | SNU-7012NT | Female/38 | Normal tissue | NA | NA |
|  | SNU-7012CT | Female/38 | Cancer tissue | NA | NA |
| 6 | SNU-7036-TO | Male/46 | Cancer organoid | 7 | 15 |
|  | SNU-7036NT | Male/46 | Normal tissue | NA | NA |
|  | SNU-7036CT | Male/46 | Cancer tissue | NA | NA |
| 7 | SNU-7057-TO | Female/55 | Cancer organoid | 6 | 9 |
| 8 | SNU-7230-TO | Female/73 | Cancer organoid | 8 | 9 |
|  | SNU-7230N-NO | Female/73 | Normal organoid | 4 | 4 |
|  | SNU-7230T | Female/73 | Cancer cell line | 2 | 2 |
|  | SNU-7230NT | Female/73 | Normal tissue | NA | NA |
|  | SNU-7230CT | Female/73 | Cancer tissue | NA | NA |
| 9 | SNU-7238-TO | Male/55 | Cancer organoid | 5 | 6 |
|  | SNU-7238T | Male/55 | Cancer cell line | 10 | 12 |
|  | SNU-7238NT | Male/55 | Normal tissue | NA | NA |
|  | SNU-7238CT | Male/55 | Cancer tissue | NA | NA |
| 10 | SNU-7375-TO | Female/77 | Cancer organoid | 3 | 8 |
|  | SNU-7375NT | Female/77 | Normal tissue | NA | NA |
|  | SNU-7375CT | Female/77 | Cancer tissue | NA | NA |
| 11 | SNU-7442-TO | Male/49 | Cancer organoid | 10 | 8 |
|  | SNU-7442NT | Male/49 | Normal tissue | NA | - |
|  | SNU-7442CT | Male/49 | Cancer tissue | NA | - |
| 12 | SNU-7490-TO | Male/67 | Cancer organoid | 8 | 10 |
|  | SNU-7490NT | Male/67 | Normal tissue | NA | NA |
|  | SNU-7490CT | Male/67 | Cancer tissue | NA | NA |
| 13 | SNU-7657-TO | Male/74 | Cancer organoid | 9 | 9 |
|  | SNU-7657T | Male/74 | Cancer cell line | 15 | 15 |
|  | SNU-7657NT | Male/74 | Normal tissue | NA | NA |
|  | SNU-7657CT | Male/74 | Cancer tissue | NA | NA |
| 14 | SNU-7687-TO | Male/76 | Cancer organoid | 5 | 9 |
|  | SNU-7687T | Male/76 | Cancer cell line | 10 | 10 |
|  | SNU-7687NT | Male/76 | Normal tissue | NA | NA |
|  | SNU-7687CT | Male/76 | Cancer tissue | NA | NA |
| 15 | SNU-7695-TO | Male/56 | Cancer organoid | 6 | 7 |
|  | SNU-7695T | Male/56 | Cancer cell line | 8 | 8 |
|  | SNU-7695CT | Male/56 | Cancer tissue | NA | NA |
| 16 | SNU-7878-TO | Male/63 | Cancer organoid | 6 | 7 |
|  | SNU-7878T | Male/63 | Cancer cell line | 13 | 13 |
|  | SNU-7878NT | Male/63 | Normal tissue | - | NA |
|  | SNU-7878CT | Male/63 | Cancer tissue | NA | NA |
| 17 | SNU-7941-TO | Male/61 | Cancer organoid | 4 | 8 |
|  | SNU-7941N-NO | Male/61 | Normal organoid | 4 | 4 |
|  | SNU-7941T | Male/61 | Cancer cell line | 11 | 11 |
|  | SNU-7941NT | Male/61 | Normal tissue | NA | NA |
|  | SNU-7941CT | Male/61 | Cancer tissue | NA | NA |
| 18 | SNU-8090-TO | Male/67 | Cancer organoid | 7 | 7 |
|  | SNU-8090NT | Male/67 | Normal tissue | NA | NA |
|  | SNU-8090CT | Male/67 | Cancer tissue | NA | NA |
| 19 | SNU-8297-TO | Male/70 | Cancer organoid | 5 | - |
|  | SNU-8297NT | Male/70 | Normal tissue | NA | NA |
|  | SNU-8297CT | Male/70 | Cancer tissue | NA | NA |
| 20 | SNU-9204-TO | Female/57 | Cancer organoid | 6 | 6 |
|  | SNU-9204N-NO | Female/57 | Normal organoid | - | 5 |
|  | SNU-9204NT | Female/57 | Normal tissue | NA | NA |
|  | SNU-9204CT | Female/57 | Cancer tissue | NA | NA |

Supplementary Table 5. The results of the R package MS.liverK.

| **SNU No.** | **Boyault** | **Chiang** | **Hoshida** | **Lee** | **Roessler** | **EPCAM.AFP** | **Consensus subtype**  **(SNU-HCC)** |
| --- | --- | --- | --- | --- | --- | --- | --- |
| SNU-6765-TO | G4 | Inflammation | S1 | A | subgroup A | NA | C2 |
| SNU-6827-TO | G4 | Inflammation | S1 | A | subgroup B | NA | C2 |
| SNU-6978-CO | G4 | Proliferation | S1 | A | subgroup A | NA | C2 |
| SNU-7002-TO | G6 | CTNNB1 | S3 | B | subgroup A | + | C1 |
| SNU-7012-TO | G4 | Proliferation | S1 | A | subgroup A | NA | C2 |
| SNU-7036-TO | G5 | CTNNB1 | S2 | B | subgroup B | NA | C1 |
| SNU-7057-TO | G1 | Unannotated | S2 | B | subgroup A | + | C1 |
| SNU-7230-TO | G2 | Inflammation | S2 | B | subgroup B | NA | C1 |
| SNU-7238-TO | G4 | Inflammation | S3 | B | subgroup B | NA | C2 |
| SNU-7375-TO | G4 | Inflammation | S1 | A | subgroup A | NA | C2 |
| SNU-7442-TO | G4 | Unannotated | S3 | B | subgroup B | NA | C1 |
| SNU-7490-TO | G2 | Unannotated | S3 | B | subgroup A | NA | C1 |
| SNU-7657-TO | G4 | Unannotated | S1 | A | subgroup A | - | C2 |
| SNU-7687-TO | G6 | CTNNB1 | S3 | B | subgroup B | NA | C1 |
| SNU-7695-TO | G5 | Proliferation | S1 | A | subgroup A | - | C2 |
| SNU-7878-TO | G3 | Inflammation | S2 | A | subgroup A | - | C2 |
| SNU-8090-TO | G5 | Proliferation | S1 | A | subgroup A | NA | C2 |
| SNU-9204-TO | G4 | Polysomy chr7 | S3 | B | subgroup B | + | C1 |

Supplementary Table 6. Concentrations of eight drugs used for drug screening.

| **Drug** | **Max concentration (µM)** | **Dilution fold** |
| --- | --- | --- |
| 5-FU | 5,000 | 1/3 |
| Oxaliplatin | 50 | 2/3 |
| Doxorubicin | 10 | 1/2 |
| Regorafenib | 50 | 1/2 |
| Everolimus | 10 | 1/2 |
| Lenvatinib | 100 | 2/3 |
| Sorafenib | 20 | 1/2 |
| Tacrolimus | 100 | 1/2 |

Supplementary Table 8.	Clinical information of patients with HBV viral markers.

| **SNU No.** | **Sex/Age** | **Liver disease** | **HBsAg** | **Anti-HBs** | **HBeAg** | **Anti-HBe** | **Anti-HBc IgG** | **HBV DNA** | **HBsAg-based status** | **HBV DNA status** |
| --- | --- | --- | --- | --- | --- | --- | --- | --- | --- | --- |
| SNU-6765 | M/57 | HBV, HCV | 5632.16 | 0 | NA | NA | NA | Not detected | Positive | Not detected |
| SNU-6827 | M/40 | HBV | 3858.43 | 0.53 | Negative | 0.01 | NA | 1510 | Positive | Detected |
| SNU-6978 | M/51 | HBV | 5288.31 | 0.04 | Negative | 0.03 | Negative | 1560 | Positive | Detected |
| SNU-7002 | M/66 | HCV, resolved HBV, Alcohol | Negative | >1000.00 | NA | NA | NA | Not detected | Negative | Not detected |
| SNU-7012 | F/38 | HBV | 6116.86 | 0.19 | NA | NA | NA | Not detected | Positive | Not detected |
| SNU-7036 | M/46 | HBV | 4585.7 | NA | Negative | Negative | NA | Not detected | Positive | Not detected |
| SNU-7057 | F/55 | HBV | 2209.35 | 0 | NA | NA | NA | Not detected | Positive | Not detected |
| SNU-7230 | F/73 | HBV | 6010.64 | 0.63 | NA | NA | NA | Not detected | Positive | Not detected |
| SNU-7238 | M/55 | HBV | 5323.52 | 0.23 | NA | NA | NA | 364 | Positive | Detected |
| SNU-7375 | F/77 | NA | Negative | 155.32 | NA | NA | NA | NA | Negative | NA |
| SNU-7442 | M/49 | HBV | 5071.32 | 0.43 | NA | NA | NA | Not detected | Positive | Not detected |
| SNU-7490 | M/67 | NA | Negative | 24.32 | NA | NA | NA | NA | Negative | NA |
| SNU-7657 | M/74 | HBV | 4905.7 | 0.22 | NA | NA | NA | 10 | Positive | Detected |
| SNU-7687 | M/76 | NA | Negative | 5.13 | NA | NA | NA | Not detected | Negative | Not detected |
| SNU-7695 | M/56 | HBV | 638.55 | 0.67 | NA | NA | NA | 19 | Positive | Detected |
| SNU-7878 | M/63 | HBV | 3691.4 | 0 | 9.25 | 0.65 | NA | 335 | Positive | Detected |
| SNU-7941 | M/61 | NA | Negative | 28.12 | Negative | NA | NA | Not detected | Negative | Not detected |
| SNU-8090 | M/67 | HBV | 3319.90 | 5.99 | Negative | NA | NA | 238000 | Positive | Detected |
| SNU-8297 | M/70 | HBV | 5175.18 | NA | NA | NA | NA | Not detected | Positive | Not detected |
| SNU-9204 | F/57 | HBV | 193.48 | 17.47 | NA | NA | NA | 11 | Positive | Detected |

Supplementary Table 9. HBV status-related DEG list.

| Total genes | | | | | 35993 |
| --- | --- | --- | --- | --- | --- |
| Differentially expressed HBV infection (Clinical data)-associated genes | | | | | |
| Type | Up | | Down | | Total |
|  | p < 0.05 | > 2.0 FC, p <0.05 | p < 0.05 | > 2.0 FC, p <0.05 |  |
| Organoid | 37 | 35 | 58 | 55 | 95 |
| Cell line | 78 | 77 | 25 | 16 | 103 |
| Tissue | 8 | 8 | 1 | 1 | 9 |
| Differentially expressed HBVSPCR-associated genes | | | | | |
| Type | Up | | Down | | Total |
|  | p < 0.05 | > 2.0 FC, p <0.05 | p < 0.05 | > 2.0 FC, p <0.05 |  |
| Organoid | 62 | 60 | 31 | 15 | 93 |
| Cell line | 95 | 87 | 34 | 27 | 129 |
| Tissue | 1 | 1 | 5 | 5 | 6 |
| Differentially expressed HBVCPCR-associated genes | | | | | |
| Type | Up | | Down | | Total |
|  | p < 0.05 | > 2.0 FC, p <0.05 | p < 0.05 | > 2.0 FC, p <0.05 |  |
| Organoid | 7 | 7 | 4 | 4 | 11 |
| Cell line | 74 | 70 | 43 | 36 | 117 |
| Tissue | 111 | 111 | 17 | 14 | 128 |
| Differentially expressed HBVXPCR-associated genes | | | | | |
| Type | Up | | Down | | Total |
|  | p < 0.05 | > 2.0 FC, p <0.05 | p < 0.05 | > 2.0 FC, p <0.05 |  |
| Organoid | 19 | 16 | 50 | 49 | 69 |
| Cell line | 8 | 8 | 20 | 18 | 28 |
| Tissue | 9 | 9 | 86 | 86 | 95 |

P-values were calculated by Wald test.
