# Supplementary material for: Integrative characterization of human hepatocellular carcinoma‐derived organoids and their HBV‐associated biology
Source: Clin Transl Med. 2026 Oct 6;16(10):e70853. doi: 10.1002/ctm2.70853 (PMC13639540; doi:10.1002/ctm2.70853)
Supplement: Supplementary file 2 [file CTM2-16-e70853-s002.zip › Supplementary figure/Supplementary Figure Legend_Revised_with tracks.docx]

Supplementary Figure 1. Establishment of HCC-derived organoids and cell lines and comparison with paired tissues. (A) Representative bright filed microscopy images and hematoxylin and eosin (H&E) staining of HCC-derived organoids and paired tissues from the patient SNU-7657, SNU-7878 and SNU-8090 (scale bars, 500μm). (B) Representative images of 13 HCC-derived cell lines (scale bars, 250μm). (C, D) Correlograms showing the Spearman correlation among (C) HCC tissues (left panel, n = 16) and adjacent non-tumor liver tissues (right panel, n = 15), and (D) HCC-derived organoids (left panel, n = 19) and adjacent non-tumor liver tissue-derived organoids (right panel, n = 3). Each circle represents the pairwise correlation between two samples, with both the color intensity and the area of the circle corresponding to the magnitude of the correlation coefficient. The color scale below indicates the correlation coefficient values ranging from 0.8 to 1.0. (E) Heatmaps showing the Spearman correlation coefficients of the mRNA expression between adjacent non-tumor liver tissue-derived organoids and paired tissues (n = 3 pairs).

Supplementary Figure 2. Consensus clustering based on mRNA expression in HCC-derived organoids. (A) The consensus matrices for k = 2, 4, and 5. The consensus matrix for k = 3 is shown in Figure 2A. The color gradient (white to dark blue) reflects the co-clustering frequency between sample pairs, with darker shades indicating higher consensus similarity. Color bars at the top and left of each matrix represent assigned cluster subgroups. (B) Tracking plot illustrating the re-allocation of individual samples across consensus cluster numbers (k = 2 to 5). Colors indicate specific cluster assignments at each k. (C) Silhouette plot of consensus subtypes identified at k = 2 (top left), k = 3 (top right), k = 4 (bottom left), and k = 5 (bottom right). The y-axis displays individual silhouette widths, where higher positive values reflect stronger intra-cluster cohesion and inter-cluster separation. Horizontal red dashed lines indicate the overall average silhouette width for each k. Colors correspond to the designated cluster groups (cluster 1-5). Among all evaluated numbers of clusters, k = 3 showed the highest clustering stability (average silhouette width = 0.85).

Supplementary Figure 3. Comparison of subtyping from 18 SNU-HCC organoids with the subtyping from previously reported signature genes. (A) Subtyping results generated using the MS.liverK R package. Top, predicted molecular subtypes for individual organoid samples across established HCC classification systems (Lee, Hoshida, Boyault, Chiang, Roessler, and EPCAM/AFP). Colors correspond to discrete subtype categories as defined in the key. Bottom, heatmap showing the scores for the subtype signatures. Red indicates higher signature scores, whereas blue indicates lower signature scores. (B-D) Heatmaps showing the expression patterns of signature genes from (B) Hoshida et al.’s study, (C) Caruso et al.’s study, and (D) Ji et al.’s study. Each column represents an individual SNU-HCC organoid sample, and each row represents a signature gene. Gene expression values are shown as Z-scores, with red and blue indicating higher and lower relative expression, respectively. The top annotation indicates the consensus cluster (k = 2) identified based on the expression profiles of the corresponding signature genes. The left annotation indicates the subtype/signature category of each gene. Detailed lists of the signature genes are provided in Table S4.

Supplementary Figure 4. Mutation status of TP53 and CTNNB1 across consensus subtypes of SNU-HCC organoids. Each column represents an individual organoid (n = 18), and each row represents a gene. The top color bar indicates the C1/C2 subtypes. Gray and black boxes represent wild-type (WT) and mutated (M) genes, respectively.

Supplementary Figure 5. Drug-response curves of SNU-HCC organoids to eight drugs. Drug-response curves were generated for 18 SNU-HCC organoid models treated with sorafenib, regorafenib, doxorubicin, tacrolimus, oxaliplatin, lenvatinib, everolimus, and 5-FU. Each curve represents an individual organoid model, as indicated in the legend. All measurements were performed in triplicate. The x-axis shows the drug concentration on a logarithmic scale, and the y-axis shows the survival fraction relative to the untreated control, expressed as a percentage. Points represent the mean survival fraction at each drug concentration, and error bars indicate SD of replicate measurements. Lines represent the fitted drug-response curves.

Supplementary Figure 6. Drug response of SNU-HCC organoids across the C1/C2 subtypes. Heatmap showing drug response of sorafenib, tacrolimus, regorafenib, oxaliplatin, doxorubicin, and 5-FU in 18 SNU-HCC organoids. Each column represents an individual SNU-HCC organoid, and the top annotation indicates the C1/C2 subtypes. The color scales represent AUC values, with the ranges indicated by the corresponding color bars. P-values indicate comparisons of AUC values between C1 and C2 subtypes and were calculated by Wilcoxon rank-sum test.

Supplementary Figure 7. Relationship between lenvatinib-sensitivity and the pyrimidine metabolism pathway. (A) STRING analysis showing protein-protein interaction of seven genes within pyrimidine metabolism pathway. (B) Left, boxplots showing mRNA expression levels of DTYMK between C1 and C2 in SNU-HCC organoids (n = 18). P-values were calculated by Wilcoxon rank-sum test. Right, scatter plots (right panels) displaying spearman correlation between lenvatinib sensitivities (AUC) and mRNA expressions across HCC organoids (n = 19, including SNU-7941-TO). The solid line represents the fitted regression line, and the shaded area represents the 95% CI.

Supplementary Figure 8. HBV status in consensus subtypes of 18 SNU-HCC organoids. Each column represents an individual organoid (n = 18), and each row corresponds to HBV clinical data, HBVSPCR, HBVCPCR, and HBVXPCR, respectively. The top color bar indicates the C1/C2 subtypes. Gray and black boxes represent negative and positive status, respectively.

Supplementary Figure 9. Association between calcium signaling, drug response and HBV infection in SNU-HCC organoids. (A) Violin plots representing GSVA enrichment scores of the GOBP_REGULATION_OF_CALCIUM_MEDIATED_SIGNALING pathway in HCC-derived organoids (n = 19) by HBV infection status in (left) HBVCPCR and (right) HBVXPCR. Colors indicate HBV-negative and HBV-positive samples. P-values were calculated by Wilcoxon rank-sum test. (B) PCA plots showing the clustering of HCC-derived organoid samples (n = 19) by HBV infection status in (left) clinical, (middle) HBVCPCR, and (right) HBVXPCR. PCA was performed using the expression profiles of 169 calcium signaling pathway-related genes from the KEGG database. (C) Heatmap showing drug response for HCC organoids (n = 18) against eight chemotherapeutic agents, which include sorafenib, regorafenib, doxorubicin, tacrolimus, oxaliplatin, lenvatinib, everolimus, and 5-FU. Each column represents an individual organoid sample, and each row represents a drug. The top annotations indicate HBV status based on clinical data, HBVSPCR, HBVCPCR, and HBVXPCR. Drug sensitivity is represented by AUC Z-scores, with red and blue indicating higher and lower AUC Z-scores, respectively. (D) Heatmap showing drug response of sorafenib, lenvatinib, regorafenib, everolimus, doxorubicin, tacrolimus, and 5-FU in 18 SNU-HCC organoids. The color annotations represent the HBV status based on clinical data, HBVSPCR, HBVCPCR, and HBVXPCR. The color scales represent AUC values, with the ranges indicated by the corresponding color bars.
